# Intratumor microbiota in cancer pathogenesis and immunity: from mechanisms of action to therapeutic opportunities

**DOI:** 10.3389/fimmu.2023.1269054

**Published:** 2023-10-06

**Authors:** Man Wang, Fei Yu, Peifeng Li

**Affiliations:** Institute for Translational Medicine, The Affiliated Hospital of Qingdao University, College of Medicine, Qingdao University, Qingdao, China

**Keywords:** tumor microenvironment, intratumoral microbiota, cancer pathogenesis, tumor immunity, cancer metabolism, microbiota-targeted therapy

## Abstract

Microbial species that dwell human bodies have profound effects on overall health and multiple pathological conditions. The tumor microenvironment (TME) is characterized by disordered vasculature, hypoxia, excessive nutrition and immunosuppression. Thus, it is a favorable niche for microbial survival and growth. Multiple lines of evidence support the existence of microorganisms within diverse types of cancers. Like gut microbiota, intratumoral microbes have been tightly associated with cancer pathogenesis. Intratumoral microbiota can affect cancer development through various mechanisms, including induction of host genetic mutation, remodeling of the immune landscape and regulation of cancer metabolism and oncogenic pathways. Tumor-associated microbes modulate the efficacy of anticancer therapies, suggesting their potential utility as novel targets for future intervention. In addition, a growing body of evidence has manifested the diagnostic, prognostic, and therapeutic potential of intratumoral microorganisms in cancer. Nevertheless, our knowledge of the diversity and biological function of intratumoral microbiota is still incomplete. A deeper appreciation of tumor microbiome will be crucial to delineate the key pathological mechanisms underlying cancer progression and hasten the development of personalized treatment approaches. Herein, we summarize the most recent progress of the research into the emerging roles of intratumoral microbiota in cancer and towards clarifying the sophisticated mechanisms involved. Moreover, we discuss the effect of intratumoral microbiota on cancer treatment response and highlight its potential clinical implications in cancer.

## 1 Introduction

The presence of bacteria within human tumors was first recognized in the 19th century (1). Due to difficulties in profiling the low biomass of microorganisms within tumors, our understanding of the link between tumor microbiota and cancer pathogenesis has made little progress during the first 50 years of the 20th century (2). In 1983, Warren et al. (3) observed that *Helicobacter pylori*, a causative agent of gastric cancer (GC), colonized the gastric epithelium. In 2006, bacteria-induced DNA damage was reported (4). Now, the advance in detection techniques is presenting unprecedented opportunities to investigate the diversity and functional features of intratumoral microbiota. Importantly, recent work has shed light on the perplexing roles of intratumoral microorganisms in cancer progression and their intercommunications with the immune system (5–7). These microbial residents can alter response to anticancer therapy and may be used as new biomarkers and therapeutic targets for cancer. Targeted manipulation of tumor microbiota will accelerate the pace to personalized medicine in cancer. However, the precise mechanisms that underlie the action of intratumoral microbiota in cancer development and treatment remain poorly understood. Gaining greater insight into the characteristics and biological functions of tumor-specific microbes would bring a new revolution in cancer management. In this review, we present the available data on the relationship between intratumoral microbes and cancer pathogenesis, and highlight the relevance of intratumoral microbiota in tumor immunity and therapeutic responses. We discuss the possibility of applying intratumoral microbiota as novel biomarkers and therapeutic targets for cancer and propose significant challenges for the development of tumor microbiota-targeted therapeutics.

## 2 Diversity of intratumoral microbiome

The bacterial composition and richness of different cancer types are highly heterogeneous, as verified by a comprehensive study of the tumor microbiome by Ravid Straussman’s team (8). They profiled the microbial compositions across seven human tumor types, including glioblastoma multiforme (GBM), bone, brain, breast, lung, ovary and pancreatic tumors. Each tumor type possessed distinctive microbiota signatures (**Table 1**). The microbial communities within breast cancer were more abundant and diverse than those in other tumor types. The bacterial load and richness were also higher in breast tumors than their adjacent normal tissues. Particularly, the composition of microbial species was different across subtypes of the same tumor type. Bacteria belonging to the phyla Firmicutes and Proteobacteria dominated the microbiota of all cancer types, while members of the phylum Proteobacteria were the most abundant bacteria in pancreatic tumors. Bacteria from the families *Corynebacteriaceae* and *Micrococcaceae* were abundant in nongastrointestinal tumors (e.g., bone and breast cancer). At the species level, *Fusobacterium nucleatum* was enriched in breast and pancreatic tumors. Intratumoral bacteria were located inside both cancer and immune cells, especially macrophages. Live bacteria from the phyla Actinobacteria, Firmicutes and Proteobacteria were found inside breast tumors. Metagenomic analyses proved that fungi including *Blastomyces gilchristii*, *Candida albicans*, *Malassezia globosa*, *Malassezia restricta* and *Saccharomyces cerevisiae* were present in various human cancer types (breast, bone, brain, colon, lung, melanoma, ovary and pancreas) (9). All tumor types possessed increased fungal loads compared with normal controls. Like bacteria, fungi mainly localized to tumor cells and macrophages. Different cancers also displayed cancer type-specific mycobiome profiles. For instance, colon cancer had a high abundance of Saccharomycetes, while the relative abundance of Malasseziomycetes was higher in melanoma. Viral infection has been intimately intertwined with solid malignancies including breast, cervical, colon, esophageal, gastric, hepatocellular, lung and oral cancers (23–27). Several viruses such as Epstein-Barr virus (EBV), hepatitis B virus (HBV), hepatitis C virus (HCV), human papilloma virus (HPV), human T-cell lymphotropic virus (HTLV) and Kaposi sarcoma herpes virus (KSHV) are identified as risk factors for carcinogenesis (28). Particularly, the causal effect of HPV on the initiation of bladder cancer, cervical cancer, esophageal squamous cell carcinoma (ESCC) and head and neck cancers has been documented (10–13). In addition to HPV, EBV and polyomavirus infections can also lead to ESCC (13). HBV and HCV act as contributing factors to the oncogenesis of liver cancer and cholangiocarcinoma (14). Remarkably, the prevalence of *Vibrio*- and *Streptococcus*-inhabiting bacteriophage communities was previously verified in the gut of patients with colorectal cancer (CRC) (29). Collectively, the widespread existence of microorganisms in cancer cells underscores their implication in cancer pathogenesis. An in-depth investigation of the composition and function of tumor microbiota will open up new therapeutic opportunities for cancer. To gain a better understanding of heterogeneity in intratumoral microecology, cancer type-specific microbial profiles will be described below.

**Table 1:** Heterogeneity of intratumoral microorganisms across different cancers.

Cancer type	Microorganisms	Relative abundance	Biological function	Reference
Breast cancer	*Actinomyces massiliensis*, *Lactobacillus iners*, *Enterobacter asburiae*, *Fusobacterium nucleatum*, Dothideomycetes, Eurotiomycetes, Malasseziomycetes, Saccharomycetes, *Cladosporium sphaerospermum*, *Malassezia restricta*	Enriched	Undetermined	(8, 9)
Breast cancer	*Malassezia globosa*	Enriched	Correlate with shorter overall survival	(9)
Bone cancer	*Actinomyces massiliensis*, *Enterobacter asburiae*, *Sphingomonas yanoikuyae*, Dothideomycetes, Eurotiomycetes, Saccharomycetes	Enriched	Undetermined	(8, 9)
Colon cancer	*Fusobacterium nucleatum*, Saccharomycetes	Enriched	Undetermined	(8, 9)
Glioblastoma multiforme	Agaricomycetes, Dothideomycetes, Malasseziomycetes	Enriched	Undetermined	(9)
Lung cancer	*Lactobacillus iners*, Dothideomycetes, Malasseziomycetes	Enriched	Undetermined	(8, 9)
Melanoma	Dothideomycetes, Malasseziomycetes, Saccharomycetes	Enriched	Undetermined	(9)
Melanoma	*Cladosporium*	Enriched	Reduce treatment response	(9)
Ovarian cancer	Dothideomycetes, Eurotiomycetes, Malasseziomycetes	Enriched	Undetermined	(9)
Ovarian cancer	*Phaeosphaeria*	Enriched	Correlate with shorter progression free survival	(9)
Pancreatic cancer	*Enterobacter asburiae*, *Fusobacterium nucleatum*, Malasseziomycetes, Saccharomycetes	Enriched	Undetermined	(8, 9)
Bladder cancer, cervical cancer, head and neck cancers	Human papillomavirus	Enriched	Contribute to carcinogenesis	(10–12)
Esophageal squamous cell carcinoma	Human papillomavirus, Epstein-Barr virus, polyomavirus	Enriched	Contribute to carcinogenesis	(13)
Liver cancer, cholangiocarcinoma	Hepatitis B virus, hepatitis C virus	Enriched	Contribute to carcinogenesis	(14)
Non-small cell lung cancer	*Alloprevotella*, *Brevundimonas*, *Escherichia-Shigella*, *Faecalibacterium*, *Pseudomonas*, *unclassified Enterobacteriaceae*, *Acinetobacter Jungii*, *Enterococcus*, *Haemophilus haemolyticus*, *Haemophilus parainfluenzae*, *Streptococcus pneumoniae*, *Actinomyces Neesii*, *Haemophilus*, *Haemophilus influenzae*, human herpes virus type 7, *Neisseria lactose*, *Prevotella II*, *Streptococcus constellatus*, *Streptococcus crista*, *Streptococcus gordonii*	Enriched	Undetermined	(15, 16)
Liver cancer	Actinobacteria, Bacteroidetes, Firmicutes, Proteobacteria, *Agrobacterium*, *Rhizobiaceae*	Enriched	Undetermined	(17)
Liver cancer	*Pseudomonas*	Decreased	Correlate with better prognosis	(17)
Colon adenocarcinoma	Pasteurellales	Enriched	Correlate with tumor stage	(18)
Colon adenocarcinoma	*Alistipes*, *Blautia*	Decreased	Correlate with better prognosis	(18)
Rectum adenocarcinoma	*Porphyromonas*	Enriched	Correlate with tumor stage	(18)
Colorectal carcinoma	*Bifidobacterium*	Enriched	Correlate with the extent of signet ring cells	(19)
Pancreatic adenocarcinoma	*Citrobacter freundii*, *uncultured Pseudomonadales bacterium* HF0500_12O04, *Acidovorax ebreus* TPSY, *Shigella sonnei* Ss046, the primary endosymbiont of *Sitophilus zeamais*, *Toxypothrix* sp. PCC 7601, *Mycoplasma hyopneumoniae*, *uncultured bacterium* HF0500_10F10	Enriched	Correlate with upregulation of oncogenic pathways, downregulation of tumor suppressive pathways, and immunosuppression	(20)
Renal cell carcinoma	*Actinomyces*, *Amycolatopsis*, *Brevundimonas*, *Deinococcus*, *Gordonia*, *Microlunatus*, *Nitriliruptor*, *Phyllobacterium*, *Pseudoclavibacter*, *Weissella*	Enriched	Undetermined	(21)
Renal cell carcinoma	Burkholderiales, *Comamonadaceae*	Decreased	Undetermined	(21)
Renal cell carcinoma	*Chloroplast*, *Klebsiella*, Streptophyta	Decreased	Differentiate RCC tissues from normal tissues	(21)
Renal cell carcinoma	*Rhodoplanes*	Decreased	Correlate with tumor stage	(21)
Cervical cancer	*Fusobacterium*, *Peptoniphilus*, *Prevotella*	Enriched	Undetermined	(22)
Cervical cancer	*Prevotella bivia*	Enriched	Promote cancer progression and correlate with poor prognosis	(22)
Cervical cancer	*Lactobacillus*	Decreased	Undetermined	(22)

### 2.1 Respiratory system tumors

Dumont-Leblond et al. (15) profiled the lung microbiota of twenty-nine patients with non-small cell lung cancer (NSCLC) using a 16S rRNA sequencing approach. *Diaphorobacter*, *Micrococcus*, *Paracoccus*, *Phascolarctobacterium* and *Ralstonia* were overabundant in both healthy and cancerous tissues in NSCLC patients. Enteric, inflammatory or pathogenic bacteria, including *Alloprevotella*, *Brevundimonas*, *Escherichia-Shigella*, *Faecalibacterium*, *Pseudomonas* and *unclassified Enterobacteriaceae*, were only detected in cancerous tissues. Another study also revealed that pathogenic microbes, such as *Acinetobacter Jungii*, *Enterococcus*, *Haemophilus haemolyticus*, *Haemophilus parainfluenzae* and *Streptococcus pneumoniae*, were prevalent in lung tumor tissues (16). The common pathogenic microbes may cause lung infections due to the compromised immune system. It is intriguing whether these bacteria lead to pulmonary carcinogenesis or whether their enrichment is a result from the alteration of the pulmonary environment in cancer patients. Intratumoral pathogens could cause direct damage or induce chronic inflammation, contributing to lung cancer pathogenesis. Future work should concentrate on unraveling the involvement of intratumoral microbiota in lung carcinogenesis. The effect of NSCLC microbiota on clinical outcomes necessitates thorough exploration. Pathogenic microbes may also shape the tumor immune microenvironment. The crosstalk between intratumoral microbiota and host immune system is worthy of further research. In addition, there was an association between intratumoral microbiota and the microbial component in the bronchoalveolar lavage fluid (BALF) (16). *Actinomyces Neesii*, *Haemophilus*, *H. haemolyticus*, *Haemophilus influenzae*, human herpes virus type 7 (HHV-7), *Neisseria lactose*, *Prevotella II*, *Streptococcus constellatus*, *Streptococcus crista*, *Streptococcus gordonii* and *S. pneumoniae* were commonly present in the tumor tissue and BALF of NSCLC patients. It is reasonable to infer that exploration of microbial diversity and abundance in the BALF could provide hints regarding the NSCLC tumor condition.

### 2.2 Digestive system tumors

The bacterial phyla Actinobacteria, Bacteroidetes, Firmicutes and Proteobacteria were abundant in tumor tissues of patients with primary liver cancer (PLC) (17). The relative abundance of *Agrobacterium* and *Rhizobiaceae* was dramatically increased in tumor tissues, while that of *Pseudomonas* was significantly reduced. Different histopathological subtypes (combined hepatocellular-cholangiocarcinoma (cHCC-CCA), intrahepatic cholangiocarcinoma (ICC) and hepatocellular carcinoma (HCC)) of PLC possessed their respective intratumoral microbial signatures. The HCC group exhibited special bacterial biomarkers (e.g., *Enterobacteriaceae*). The microbial biomarkers may provide a potential tool for the subtype stratification of PLC. Nevertheless, more clinical studies are warranted to track intratumoral microbiota dynamics in PLC patients. Upper and lower gastrointestinal tumors had different microbiota signatures (18). The five phyla Actinobacteria, Bacteroidetes, Firmicutes, Fusobacteria and Proteobacteria dominated the microbial composition in upper gastrointestinal tumors including esophageal carcinoma (ESCA) and stomach adenocarcinoma (STAD). The level of Bacteroidetes was higher while that of Firmicutes was lower in lower gastrointestinal tumors including colon adenocarcinoma (COAD) and rectum adenocarcinoma (READ) than upper gastrointestinal tumors. The Bacteroidetes/Firmicutes ratio was higher in lower gastrointestinal tumors than that in upper gastrointestinal tumors. The genera *Capnocytophaga* and *Helicobacter* were more abundant in upper gastrointestinal tumors than lower gastrointestinal tumors. *Faecalibacterium* and *Porphyromonas* were enriched in READ. The high levels of intratumoral *Alistipes* and *Blautia* correlated with better survival probability in COAD patients. The high abundances of Pasteurellales and *Porphyromonas* were associated with the tumor stage in COAD and READ, respectively. Profiling tumor-resident microbiota can be helpful in discovering prospective microbial biomarkers for cancer progression and prognosis. A great deal of further work needs to be done to elucidate the contribution of dysregulated microbiota in the pathogenesis of gastrointestinal cancer. Members of the genus *Bifidobacterium* were detected in CRC tissues (19). The amount of intratumoral *Bifidobacteria* DNA was associated with the extent of signet ring cells, alluding to a possible role of *Bifidobacteria* in regulating tumor microenvironment (TME) and tumor differentiation during CRC progression. *Bifidobacteria*, a natural proportion of the intestinal flora, are producers of lactic acid and acetate (30). The impairment of intestinal barrier function might lead to the entry of *Bifidobacteria* into CRC tissues (31). In the gut, *Bifidobacteria* prevented colorectal carcinogenesis (32). Nevertheless, *Bifidobacteria* seemed to behave oppositely in the TME. It was likely that *Bifidobacteria*-produced lactic acid and acetate served as an energy source for CRC cells, allowing for cancer growth and immune escape. The divergent roles of *Bifidobacteria* during CRC development remain to be explored in the future.

Intratumor pancreatic microbiome was characterized through large-scale sequencing data from The Cancer Genome Atlas (TCGA) (20). Members of the phylum Proteobacteria represented the dominant bacteria in pancreatic adenocarcinoma (PAAD) and were associated with tumor metastasis. The abundance of *Citrobacter freundii*, *uncultured Pseudomonadales bacterium* HF0500_12O04, *Acidovorax ebreus* TPSY, *Shigella sonnei* Ss046, the primary endosymbiont of *Sitophilus zeamais*, *Toxypothrix* sp. PCC 7601, *Mycoplasma hyopneumoniae* and *uncultured bacterium* HF0500_10F10 exhibited an association with the upregulation of oncogenic pathways, the downregulation of tumor-suppressive pathways, and immunosuppression. The high abundance of *A. ebreus* was linked with decreased levels of M2 macrophages, memory T cells and CD8^+^ T cells. Altogether, these pancreatic microbes might be involved in PAAD occurrence and development by reprograming the TME. It is still elusive how microbes colonize the pancreas. Permeability of intestinal barrier may be a cause of bacterial dissemination into the pancreatic duct, which requires further corroboration. Intratumoral microbes could affect tumor susceptibility to chemotherapy and patient outcome. Gammaproteobacteria acted to induce gemcitabine resistance via the bacterial enzyme cytidine deaminase (33). Oppositely, *Bifidobacterium* potentiated the efficacy of immune checkpoint inhibitor (ICI) (34). It is plausible to propose that antibiotic/probiotic administration in a tumor type-specific manner could sensitize cancer cells to anticancer treatments. More extensive studies will be required to evaluate the therapeutic benefits of microbial manipulation combined with conventional treatments in cancer patients.

### 2.3 Urogenital system tumors

The compositions of bacterial communities differed between renal cell carcinoma (RCC) tissues and adjacent normal tissues (21). The species diversity was reduced in RCC tissues compared to normal tissues. The phyla Proteobacteria (64.97%) and Firmicutes (14.09%) dominated the microbiota composition of RCC tissues. The levels of Burkholderiales and *Comamonadaceae* were lower in RCC tissues than those in normal tissues. The relative abundance of *Actinomyces*, *Amycolatopsis*, *Brevundimonas*, *Deinococcus*, *Gordonia*, *Microlunatus*, *Nitriliruptor*, *Phyllobacterium*, *Pseudoclavibacter* and *Weissella* was increased in RCC tissues compared with normal tissues. *Deinococcus* was considered as a factor contributing to GC development (35). The role of *Deinococcus* in RCC is worthy of more detailed investigation. Moreover, reduced levels of *Chloroplast*, *Klebsiella* and Streptophyta could differentiate RCC tissues from normal tissues with high sensitivity and specificity. The functional analysis of RCC-associated microbiota showed that pathways responsible for cell growth and death, membrane transport and transcription were enriched in RCC tissues. Dysregulated microbes might promote RCC development by regulating these intracellular pathways, which needs to be further deciphered. Substantial research efforts should be direct toward illustrating the genuine association between intratumor microbiota and RCC pathology.

The microbes belonging to the families *Bacillaceae*, *Halobacteriaceae* and *Prevotellaceae* were abundant in cervical cancer tissues from 121 patients (22). Cervical cancer tissues had increased levels of *Fusobacterium*, *Peptoniphilus* and *Prevotella* and decreased levels of *Lactobacillus* relative to healthy uterine cervix and vagina. Co-incubation experiments showed that *Prevotella bivia* increased the expression of three immunoregulatory proteins lysosomal associated membrane protein 3 (LAMP3), signal transducer and activator of transcription 1 (STAT1) and antigen peptide transporter 1 (TAP1) in cervical cancer cells under hypoxic conditions. LAMP3 acted as a tumor promoter contributing to cervical cancer metastasis (36). It is assumed that *P. bivia* could infiltrate cervical cancer tissues and propel cervical carcinogenesis through regulation of immunological pathways. This study suggested an indirect communication between cervical microbiota and cancer cells. It is intriguing whether intratumoral microbiota can affect the functionality of infiltrating immune cells. However, additional studies are necessary to decipher the host-microbiota interaction in cervical cancer.

## 3 The origin of intratumoral microbiota

**Figure 1:**
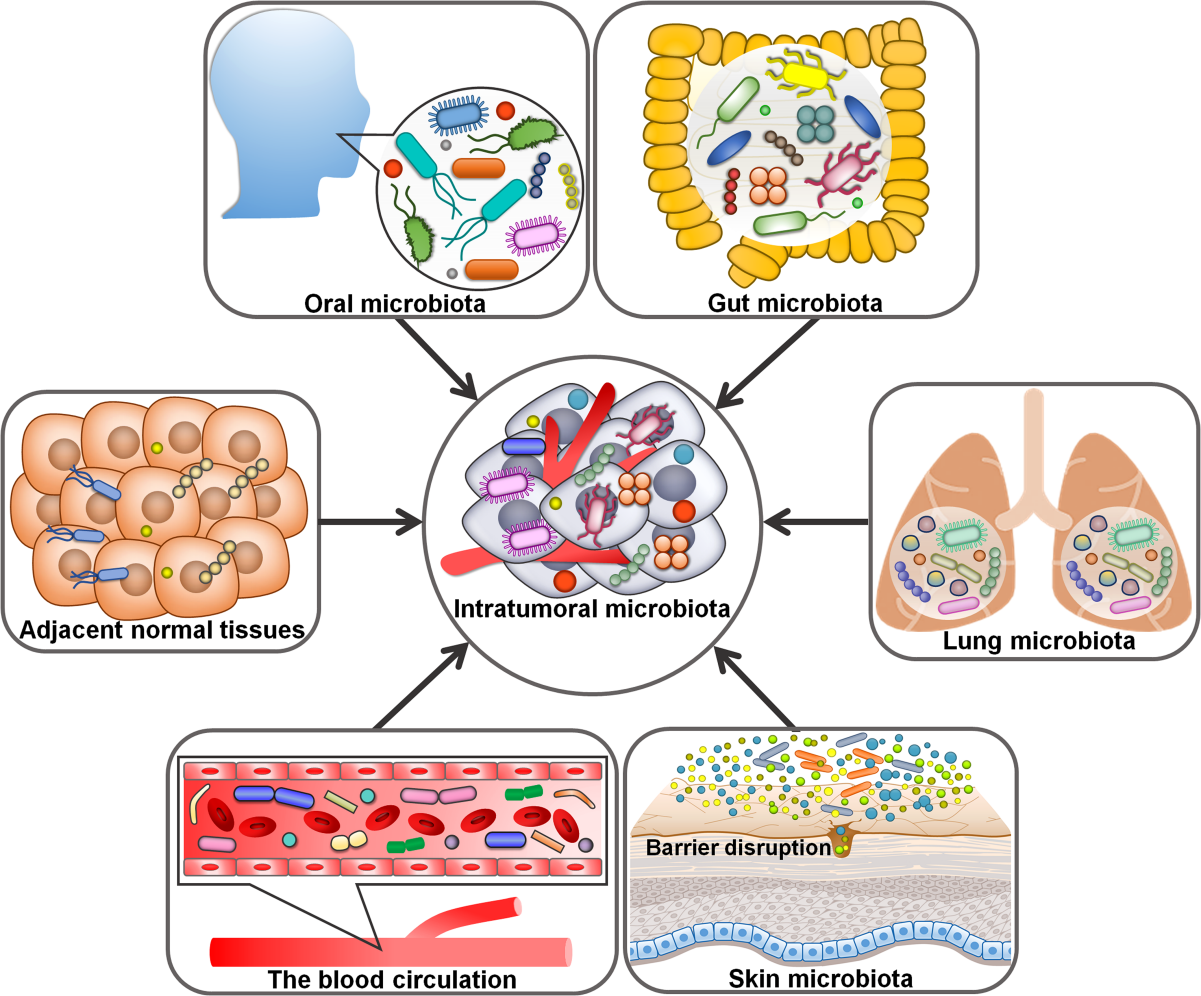
Potential origins of intratumoral microbiota. Oral and intestinal microbiota may be potential sources of intratumoral microbiota. The hypoxic and immunosuppressive tumor microenvironment could facilitate microbial migration from adjacent normal tissues to tumor tissues. For microorganisms intruding into the blood circulation from different loci, the chemotactic gradient or cellular debris released from necrotic tumor cells may support their transfer to the tumor microenvironment. Given abundant microbes exist in the mucosal organs (e.g., skin and lung), disruption in mucosal barriers may contribute to the entrance of intratumoral microorganisms.

Despite intratumoral microorganisms have attracted a great deal of attention, their origins remain a mystery. It is conceived that intratumoral microbes come from oral and intestinal microbiota, adjacent normal tissues, the circulatory system and mucosal sites (**Figure 1**). Compelling evidence has indicated that intestinal microbiota could be a main source of intratumoral microbes (37). Intestinal microbes can be detected in digestive system tumors (e.g., CRC and liver cancer), lung cancer and cervical cancer. All these organs have an externally exposed cavity, providing conditions propitious for microbial colonization. Microorganisms residing in the intestine may invade these tumor tissues owing to the permeability of intestinal barrier during carcinogenesis. A bacterial driver-passenger model was previously proposed (31). Specifically, intestinal bacteria such as *Bacteroides fragilis* and *H. pylori* acted as drivers of carcinogenesis and contributed to the creation of an appropriate condition that enabled the transfer of “passenger” bacteria, especially opportunistic bacteria, to the TME. Intestinal microbes are likely to play an important role in microbial intrusion into the TME. Considering the similar microbial composition between tumor tissues and their adjacent normal tissues, it is presumed that adjacent normal tissues are a potential source of intratumoral microbiota (37). The hypoxic and immunosuppressive TME favors microbial growth, which may be an explanation for microbial migration from noncancerous tissues to cancerous tissues. More research is required to clarify how microbes transfer from adjacent normal tissues to the tumor site. It is worth noting that the source of microbes within normal tissues remains ambiguous. Conversely, microorganisms inhabiting the tumor tissues may enter the normal tissues. This hypothesis requires further validation.

Given the abundant and aberrant vascular architecture in tumors, oral and intestinal microorganisms could travel through the circulation system and enter distant tumor tissues via damaged blood vessels (38). For instance, the oral bacterium *F. nucleatum* could gain access to CRC tissues via the hematogenous route and colonize there (39). *Escherichia coli* impaired intestinal vascular barriers and migrated to the liver via the blood circulation, where it promoted the creation of a pre-metastatic microniche (40). It is not excluded that microbial species in the circulation system may directly invade the tumor tissues. Within the tumor tissue, microbes may be directly uptaken by tumor cells (41). For microbes intruding into the blood circulation from diverse loci, the chemotactic gradient or necrotic cell-released debris in tumors may be the cause of their transfer to the TME. Massive microbes exist in the mucosal organs including the skin and lung, and disruption in mucosal barriers may give rise to the entrance of intratumoral microbes (42). That is to say, intratumoral microbiota may originate from mucosal sites. Tumor-associated microorganisms are predominantly present in cancer cells and immune cells, implying that microbes may be delivered to the tumor site in the form of fractions or intact cells through cell migration (9, 43). Erythrocytes were considered as potential carriers for live bacteria into the tumors (44). Altogether, intratumoral microorganisms have various sources and show a close relationship with oral and intestinal microbiota. The mechanisms by which microorganisms enter the tumors are multitudinous and varied. Future work should be directed toward corroborating the diverse origins of intratumoral microbiota and elucidating the mechanisms regulating microbial translocation into the tumor site. A deeper understanding of the sources of intratumor microbes would provide new perspectives and theoretical basis for cancer prevention and treatment.

## 4 The mechanisms of action of intratumoral microbiota in cancer

### 4.1 Mechanisms of intratumoral microbiota affecting cancer development

**Figure 2:**
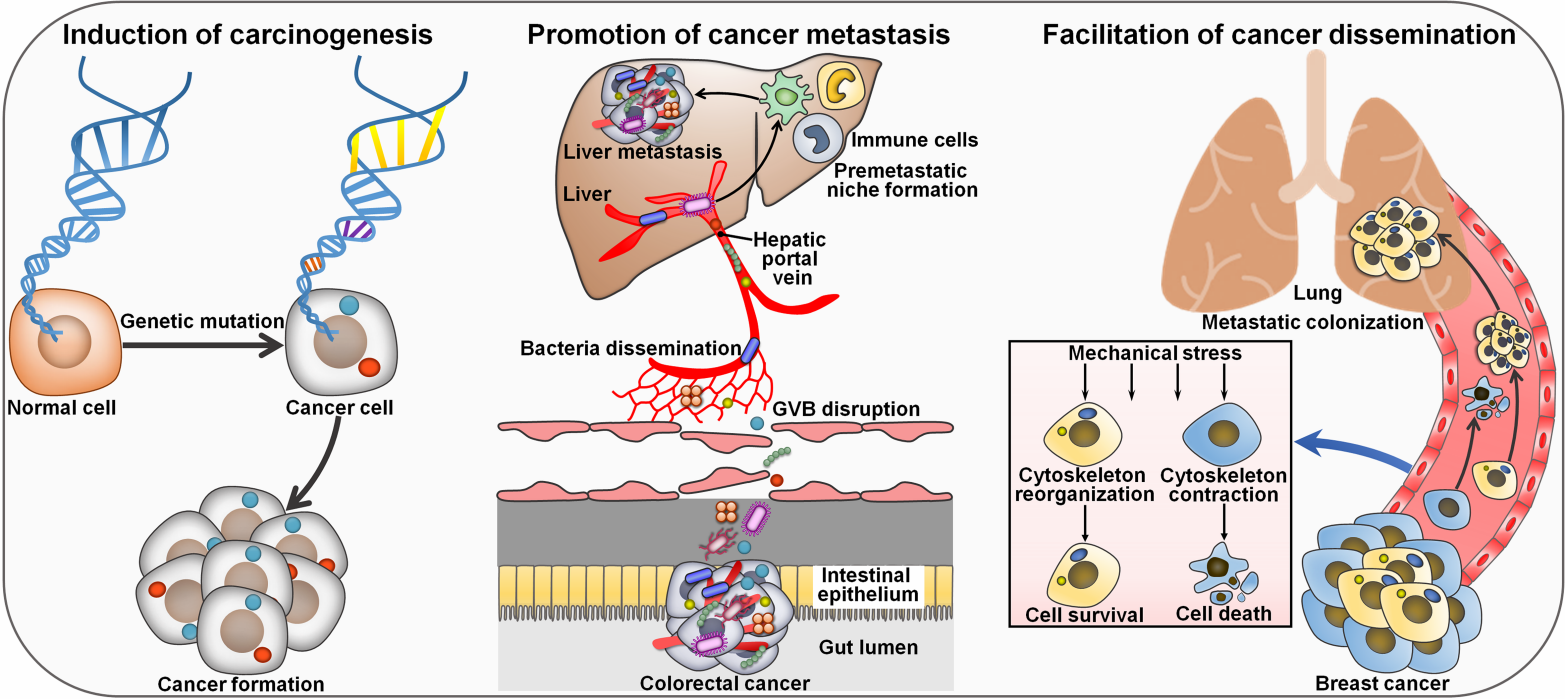
Mechanisms of intratumor microbiota affecting cancer pathogenesis. Intratumoral microorganisms act as a tumor inducer by causing genetic mutation in host cells. Colorectal cancer-resident microbes can impair the GVB and disseminate to the liver where they recruit immune cells (e.g., macrophages, neutrophils and inflammatory monocytes). These events lead to the formation of a pre-metastatic microenvironment that promotes cancer metastasis to the liver. Intratumoral microbes reorganize actin cytoskeleton in circulating breast cancer cells and foster their survival against mechanical stress in the circulation, culminating in enhanced lung metastasis. GVB, gut vascular barrier.

Intratumoral microbes have been identified as key regulators of cancer progression (**Table 2**). Liu et al. (45) characterized the microbial communities within CRC or precancerous adenoma. The levels of CRC-associated genera *Bacteroides*, *Fusobacterium*, *Parvimonas* and *Prevotella* were markedly different between CRC and adenoma tissues. The abundance of these genera varied across different sites of a single neoplasia, indicating the heterogeneity of microbial communities within a single CRC or nonmalignant adenoma. The amount of intra-neoplasia microbes with high variation such as Proteobacteria diminished along the adenoma-carcinoma sequence, suggesting the progressive alteration in abundance and diversity of microbes in the course of colorectal carcinogenesis. Firmicutes species (e.g., *Clostridium*, *Parvimonas* and *Peptostreptococcus*) were the most abundant members that displayed strong association with Kirsten rat sarcoma viral oncogene homologue (KRAS) mutation in CRC tumors and adenomas. Genera belonging to Proteobacteria (e.g., *Dechloromonas* and *Gallionella*) were the most abundant microbes that positively correlated with microsatellite instability (MSI) in CRC patients. The majority of intratumoral microbes that were related to CRC-associated genetic markers involving KRAS mutation and MSI showed high variation in abundance. The interplay between gut microbiota and host genetic mutation could accelerate cancer pathogenesis (68) (**Figure 2**). Host genetic factors in turn alter the microbial composition (69). More attention should be paid to the complex interrelation between gut microbiota and host genetic factors. Intratumoral microbiota heterogeneity may affect the metabolism of precancerous or cancer cells. Additional studies are suggested to elucidate the capricious compositional and functional profiles of intra-neoplasia microbiota as well as its contribution to colorectal carcinogenesis.

**Table 2:** Summary of the effects of intratumoral microbes on cancer pathogenesis or treatment.

Cancer type	Microorganism	Role	Effect on cancer progression/treatment	Reference
Colorectal cancer	*Bacteroides*	Pathogenic microbe	Correlate with carcinogenesis	(45)
Colorectal cancer	*Fusobacterium*	Opportunistic pathogen		
Colorectal cancer	*Parvimonas*	Commensal microbe		
Colorectal cancer	*Prevotella*	Pathogenic microbe		
Colorectal cancer	*Clostridium*	Pathogenic microbe		
Colorectal cancer	*Parvimonas*	Commensal microbe		
Colorectal cancer	*Peptostreptococcus*	Commensal microbe		
Colorectal cancer	*Dechloromonas*	Noncommensal microbe		
Colorectal cancer	*Gallionella*	Noncommensal microbe		
Colorectal cancer	*Escherichia coli C17*	Pathogenic microbe	Promote cancer metastasis	(40)
Breast cancer	*Lactobacillus animalis*	Commensal microbe	Promote cancer metastasis	(46)
Breast cancer	*Streptococcus cuniculi*	Commensal microbe		
Breast cancer	*Staphylococcus xylosus*	Commensal microbe		
Colorectal cancer	*Fusobacterium nucleatum*	Commensal microbe	Enhance pro-tumoral immunity; favor cancer progression	(47)
Pancreatic cancer	*Porphyromonas gingivalis*	Pathogenic microbe	Induce tumor-promoting inflammation; facilitate cancer growth	(48)
Pancreatic ductal adenocarcinoma	*Alternaria alternata*	Pathogenic microbe	Enhance pro-tumoral immunity; favor cancer development	(49)
Pancreatic ductal adenocarcinoma	*Malassezia globosa*	Pathogenic microbe		
Oral squamous cell carcinoma	*Fusobacterium nucleatum*	Commensal microbe	Inhibit pro-tumoral immunity; correlate with favorable clinical outcomes	(50)
Soft tissue sarcoma	*Respirovirus*	Pathogenic microbe	Increase antitumor immunity	(51)
Melanoma	*Neospora caninum*	Pathogenic microbe	Increase antitumor immunity; foster cancer cell death	(52)
Pancreatic ductal adenocarcinoma	*Pseudoxanthomonas*	Non-pathogenic microbe	Trigger antitumor immunity	(53)
Pancreatic ductal adenocarcinoma	*Saccharopolyspora*	Noncommensal microbe		
Pancreatic ductal adenocarcinoma	*Streptomyces*	Pathogenic microbe		
Lung adenocarcinoma	*Anabaena* sp. *K119*	Noncommensal microbe	Block antitumor immunity	(54)
Lung adenocarcinoma	*Uncultured bacterium*	Unknown		
Lung squamous cell carcinoma	*Pseudomonas putida str. KT2440*	Non-pathogenic microbe		
Lung squamous cell carcinoma	*Thermostaphylospora chromogena*	Noncommensal microbe		
Colorectal cancer	*Fusobacterium nucleatum*	Commensal microbe	Induce tumor-promoting inflammation; facilitate cancer invasion	(55)
Colon cancer	*Eubacteria rectale/Roseburia*	Commensal microbe	Block antitumor immunity	(56)
Gastric cancer	*Methylobacterium*	Commensal microbe	Block antitumor immunity	(57)
Pancreatic ductal adenocarcinoma	*Bifidobacterium pseudolongum*	Commensal microbe	Block antitumor immunity	(7)
Intrahepatic cholangiocarcinoma	*Paraburkholderia fungorum*	Opportunistic pathogen	Regulate cancer cell metabolism; repress cancer development	(58)
Lung cancer	*Akkermansia muciniphila*	Commensal microbe	Regulate cancer cell metabolism; repress cancer development	(59)
Epithelial ovarian cancer	*Propionibacterium acnes*	Commensal microbe	Activate the Hh signaling cascade; facilitate carcinogenesis	(60)
Papillary thyroid carcinoma	*Metschnikowia santaceciliae*	Noncommensal microbe	Inhibit the PI3K/Akt signaling pathway; correlate with cancer progression	(61)
Papillary thyroid carcinoma	*Pacynthium nigrum*	Unknown		
Papillary thyroid carcinoma	*Spriromyces aspiralis*	Unknown		
Papillary thyroid carcinoma	*Thanatephorus cucumeris*	Pathogenic microbe		
Papillary thyroid carcinoma	*Brevicellicium exile*	Unknown	Activate the p53 signaling pathway; correlate with cancer progression	
Papillary thyroid carcinoma	*Eremascus albus*	Unknown		
Papillary thyroid carcinoma	*Zoophthora occidentalis*	Pathogenic microbe		
Papillary thyroid carcinoma	*Uncultured Glomus*	Unknown	Activate the MAPK signaling pathway; correlate with cancer progression	
Colorectal cancer	*Escherichia coli*	Commensal microbe	Enhance 5-FU resistance	(62)
Colorectal cancer	*Bifidobacterium*	Commensal microbe	Improve the efficacy of anti-CD47 treatment	(63)
Breast cancer	*Megasphaera*	Commensal microbe	Improve the efficacy of anti-PD-1 treatment	(64)
Melanoma	*Bifidobacterium*	Commensal microbe	Improve the efficacy of anti-PD-L1 treatment	(65)
Gastrointestinal cancer	*Eubacterium*	Commensal microbe	Improve the efficacy of anti-PD-1/PD-L1 treatment	(66)
Gastrointestinal cancer	*Lactobacillus*	Commensal microbe		
Gastrointestinal cancer	*Streptococcus*	Pathogenic microbe		
Fibrosarcoma	*Bacteroides fragilis*	Commensal microbe	Improve the efficacy of CTLA-4 blockade	(67)

The liver is physiologically connected to the gut via the hepatic portal vein and is considered as the predominant organ site of primary CRC metastasis. However, gut vascular barrier (GVB) forms a critical barrier restricting intestinal bacteria dissemination. The high level of plasmalemma vesicle-associated protein-1 (PV-1) in primary tumors, a marker of disrupted GVB, was found to correlate with distant liver metastasis and poor prognosis in CRC patients (40). Importantly, CRC patients with high PV-1 expression in primary tumors possessed an increased amount of bacteria in metastatic liver lesions compared with paired healthy hepatic parenchyma, suggesting potential microbiota translocation through impaired GVB. Mechanistic investigation indicated that CRC-resident bacteria *E. coli* C17 directly broke down the GVB and caused PV-1 upregulation through a type III secretion system (TTSS) virulence factor (Virf)-dependent mechanism. Once GVB dysfunction, intestinal bacteria could disseminate to the liver where they fostered the recruitment of immune cells including macrophages, neutrophils and inflammatory monocytes. These events resulted in the creation of a pre-metastatic microenvironment that drove cancer metastasis to the liver. These findings revealed that intestinal bacteria targeted vascular gatekeepers to migrate into the secondary organ, contributing to the establishment of a favorable immune niche that supports the growth of disseminated cancer cells. PV-1 might act as a prognostic biomarker of gut vascular impairment and CRC distant recurrence, leading to liver metastasis formation. The clinical relevance of *E. coli* C17 for CRC progression should be further addressed. Furthermore, some beneficial bacteria (e.g., *Lactobacillus paracasei*) had the ability to restore the GVB. Therefore, there is a necessity of further research to dissect the functional link between intestinal bacteria and the metastatic process in CRC.

Spontaneous murine breast tumor displayed an expansion of commensal microorganisms that included *Enterococcus*, *Lactobacillus*, *Staphylococcus* and *Streptococcus* (46). The intratumoral microbes mainly colonized in tumor cell cytoplasm. Intracellular bacteria (e.g., *Staphylococcus xylosus*) together with host tumor cells, could travel through the circulation system and reside in distal organs. The invasion of various bacteria (e.g., *Lactobacillus animalis*, *Streptococcus cuniculi* and *S. xylosus*) promoted lung metastasis without affecting primary tumor growth. The intracellular bacteria reorganized actin cytoskeleton in circulating tumor cells and promoted host tumor cell survival by enhancing resistance to mechanical stress in the circulation system during metastatic colonization. Depletion of tumor-resident microbes suppressed the metastasis of breast tumor cells *in vivo*. Aerobic bacteria were increased in the lung metastasis, while the facultative anaerobes were decreased, implying a dynamic oxygen microenvironment in the tumor. The human and murine breast tumors possessed a similar microbial community profile, as characterized by high amounts of *Enterococcus* and *Streptococcus*. It was plausible to infer that intratumoral microbiota played an important role in the pathogenesis of human breast cancer. Intratumoral microbes may be an integral constituent of the tumor and act as pivotal drivers of tumor progression. Intratumoral microbiota could be a prospective target for better breast cancer management. It remains ambiguous how the bacteria enter tumor cells. The role of intratumor bacteria in tumor cell intravasation, extravasation and dissemination needs to be adequately delineated. Whether intratumoral microbiota, gut microbiota and the immune system cooperatively act to regulate cancer progression is an important question that emerges.

### 4.2 The crosstalk between intratumoral microbiota and tumor immunity

#### 4.2.1 Promotion of pro-tumoral immunity

**Figure 3:**
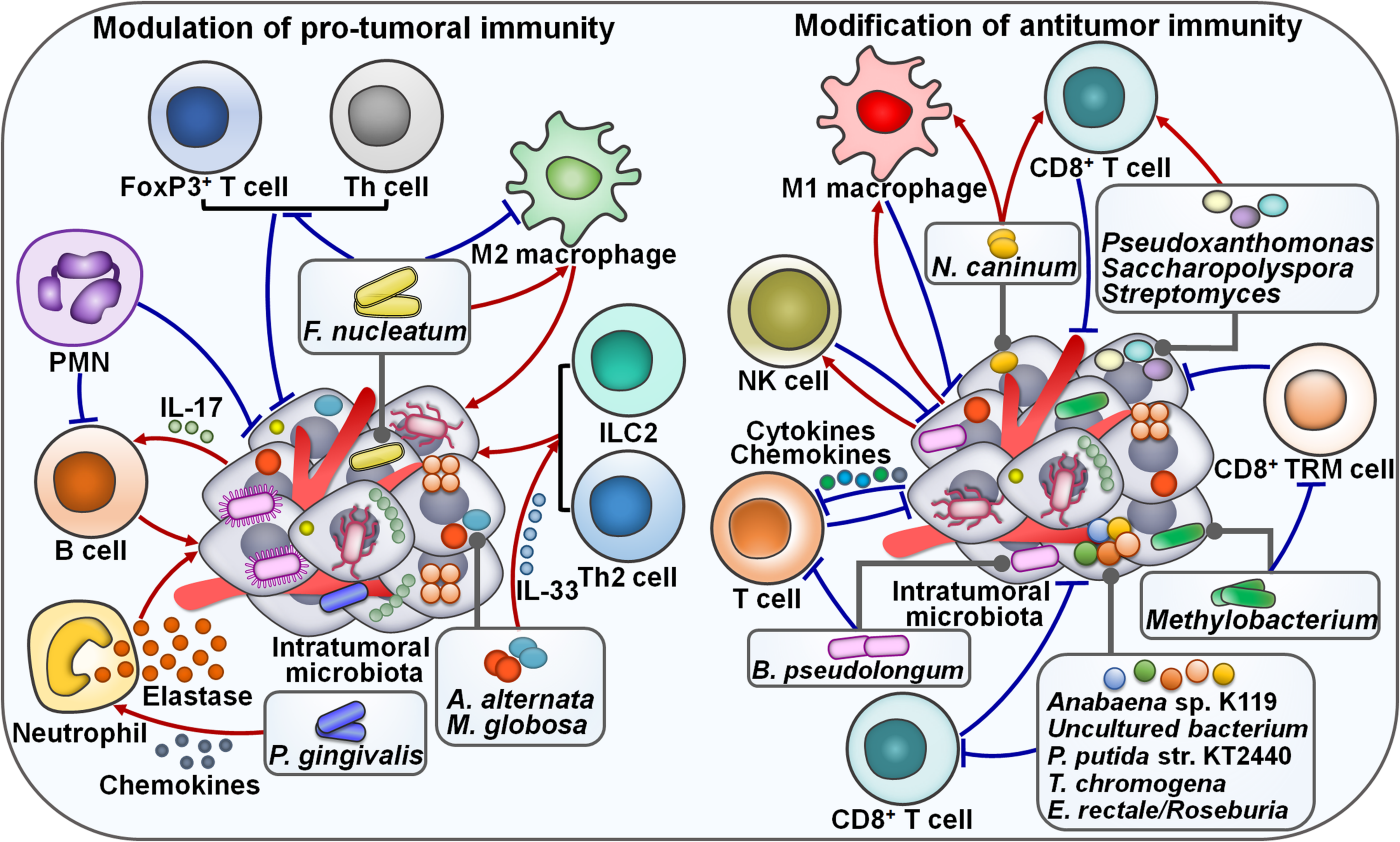
Emerging roles of intratumoral microbiota in tumor immunity. On the one hand, tumor-derived microbes are able to regulate pro-tumoral immunity. For instance, the *F nucleatum* load is associated with the population of M2 macrophages. The effects of *F nucleatum* on M2 macrophage activation vary depending on the tumor type. Intratumoral microbiota enhances IL-17 production to promote the infiltration of B cells into the tumor tissues, leading to cancer progression. PMNs restrict the expansion of tumor microbiota and suppress B cell infiltration through regulatory actions on IL-17. Intratumoral fungi *A alternata* and *M. globosa* recruit type 2 immune cells (e.g., ILC2s and Th2 cells) into the tumor microenvironment by increasing IL-33 release. Intratumoral *P. gingivalis* attracts tumor-associated neutrophils by increasing the secretion of neutrophilic chemokines. *P. gingivalis* also stimulates the secretion of neutrophil elastase from tumor-associated neutrophils, hence promoting cancer progression. On the other hand, tumor-derived microbiota plays an important role in modulating antitumor immune cell function. *N. caninum* correlates with the infiltration levels of macrophages and CD8^+^ T cells. Likewise, *Pseudoxanthomonas*, *Saccharopolyspora* and *Streptomyces* are related to the increased level of CD8^+^ T cells. The abundance of intratumoral microbiota shows a positive relationship with NK cell infiltration. Intratumor *Methylobacterium* reduces the frequency of CD8^+^ TRM cells within the tumor microenvironment. Intratumoral microbiota causes T cell exclusion by enhancing the secretion of specific chemokines and interleukins into the surrounding milieu. Particularly, *B pseudolongum* is shown to restrain T cell immunity. *Anabaena* sp. K119, *uncultured bacterium*, *P. putida* str. KT2440, *T. chromogena* and *E rectale*/*Roseburia* have a negative relationship with CD8^+^ T cells. FoxP3, forkhead box P3; Th cell, T helper cell; PMN, polymorphonuclear neutrophil; IL-17, interlekin-17; ILC2, type 2 innate lymphoid cell; IL-33, interlekin-33; NK cell, natural killer cell; TRM cell, tissue-resident memory T cell.

Increasing evidence indicates that intratumoral microbiota has a role in remodeling tumor immune microenvironment. Intratumoral microecology contributes to the recruitment and activation of tumor-supportive immune cells. Intratumoral microbiota induced interleukin-17 (IL-17) production to support the infiltration of B cells into the tumor tissues, culminating in colon cancer progression (70) (**Figure 3**). Polymorphonuclear neutrophils (PMNs), highly abundant immune cells in colon cancer, could reverse microbial dysbiosis in colon cancer tissues by reducing the amount of tumor-associated *Akkermansia* and increasing the amount of Proteobacteria. On the contrary, the absence of PMNs facilitated the outgrowth of colon microbiota and tumor-associated DNA damage. Depletion of colon microbiota or IL-17 inhibition reversed the pro-tumor effect of PMN deficiency. Collectively, PMNs limited colon cancer development by restraining the expansion of colon microbiota and reducing B cell infiltration through IL-17. It was possible that tumor-infiltrating PMNs evolved an immunosuppressive phenotype throughout colon cancer development (71). The perplexing contribution of PMNs in colon cancer progression deserves special attention. The mechanisms through which PMNs restrict colon microbiota expansion need to be further revealed. The high load of *F. nucleatum* inside MSI-high CRCs was markedly associated with tumor growth and invasion (47). *F. nucleatum*-enriched subset of MSI-high CRCs had decreased forkhead box P3 (FoxP3)^+^ T cells at both invasive margin and center of tumor areas. High load of *F. nucleatum* showed a positive relationship with an elevated ratio of CD163^+^ macrophages to CD68^+^ macrophages in MSI-high CRCs, alluding to increased population of M2-polarized macrophages in the tumor center. Thus, *F. nucleatum* might be connected with pro-tumoral immunity in MSI-high CRCs. Reducing *F. nucleatum* level could be effective in inhibiting CRC progression, which necessitates further study.

*Porphyromonas gingivalis*, a pathogen of periodontitis, was more abundant in pancreatic cancer (PC) tissues than in normal adjacent tissues, implying its momentous role in PC pathogenesis (48). Gavage with *P. gingivalis* accelerated tumor growth in PC-bearing mice. Remarkably, *P. gingivalis* was enriched in the tumor tissues of PC mice. *P. gingivalis*-treated PC tissues displayed a neutrophil-mediated proinflammatory TME. Mechanistically, intratumoral *P. gingivalis* recruited tumor-associated neutrophils by increasing the secretion of neutrophilic chemokines (C-X-C motif chemokine ligand 1 (CXCL1), CXCL2, C-X-C motif chemokine receptor 2 (CXCR2), IL-17F, S100a8 and S100a9). After that, *P. gingivalis* fostered the secretion of neutrophil elastase from tumor-associated neutrophils to drive PC development. The mechanism by which *P. gingivalis* enhances the secretion of neutrophil extracellular trap (NET)-associated proteases needs to be elucidated. *P. gingivalis* was commonly present in both the oral and intratumoral microbiota, implying the potentially bacterial migration from the oral cavity to the pancreas. The existence of *P. gingivalis* in the faeces of PC mice provided evidence of an oral-gut-pancreas translocation route. Inhibition of *P. gingivalis* infection may be beneficial for PC intervention. Future follow-up work is recommended to explore the mechanistic link between *P. gingivalis* colonization and the proinflammatory microenvironment in PC. It will be important to clarify whether other intratumoral pathogens can exert the same effect. The impact of intratumoral microbes on tumor immunity extends beyond bacterial species. Mycobiota has also been associated with cancer progression. Pancreatic ductal adenocarcinoma (PDAC) TME was infiltrated with type 2 immunocytes including T helper 2 (Th2) cells and type 2 innate lymphoid cells (ILC2s) (49). Notably, PDAC tumors contained a higher abundance of fungal communities than the normal pancreas. Intratumoral fungi *Alternaria alternata* and *M. globosa* actuated the dectin-1 signaling pathway in cancer cells that promoted *Kras* ^G12D^-mediated IL-33 release. The released IL-33 recruited and induced type 2 immune cells into the tumor environment and facilitated PDAC development. As expected, *in vivo* experiment indicated that anti-fungal treatment or IL-33 deficiency decreased the infiltrating level of Th2 cells and ILC2s, leading to tumor regression and increased survival in PDAC-bearing mice. Retrograde transfer via the opening of the sphincter of Oddi might be a route for microbial migration from the duodenum to the pancreas. It is necessary to determine when the retrograde transfer of fungi occurs during the process of PDAC development. Continued research efforts are required to investigate whether intratumoral bacteria act synergistically with fungal communities to regulate host immune response and promote PDAC growth. ILC2s play divergent roles at different stages of cancer development. The inducing effects of intratumoral fungi on the IL-33/ILC2 axis potentially have variable consequences according to the cancer type, location and the state of the TME. The complex roles of intratumoral fungal communities in the occurrence and development of cancer should be a research priority in the future.

#### 4.2.2 Inhibition of pro-tumoral immunity

On the contrary, intratumoral microbiota also acts to counteract pro-tumoral immune responses. Reportedly, patients with *F. nucleatum*-positive oral squamous cell carcinoma (OSCC) had a lower recurrence rate, less frequent lymph node invasion and metastatic relapse than patients with *F. nucleatum*-negative tumors (50). Moreover, *F. nucleatum*-positive cases showed a trend toward longer overall survival (OS), better relapse-free survival (RFS) and metastasis-free survival (MFS) than *F. nucleatum*-negative cases. These findings demonstrated the prognostic significance of *F. nucleatum* in OSCC. The *F. nucleatum* load inversely correlated with the markers of B lymphocytes, fibroblasts, M2 macrophages and Th lymphocytes. Meanwhile, high *F. nucleatum* level was correlated with the decreased expression of OX40 ligand (tumor necrosis factor superfamily member 4 (TNFSF4)) and Toll-like receptor 4 (TLR4). The expression level of TNFSF9 receptor (TNFRSF9) was reduced while that of TNFSF9 and IL-1β was increased in OSCC tissues with high *F. nucleatum* load. *F. nucleatum* exerted both proinflammatory and immunosuppressive effects (72, 73). It was likely that OSCC-associated *F. nucleatum* remodeled the TME and rendered it insensitive to proinflammatory signals, as shown by the downregulation of TLR4 and M2 macrophages, thus leading to favorable clinical outcomes. Randomized clinical studies should be carried out to substantiate the interplay between *F. nucleatum* and tumor immune microenvironment. It will be equally important to examine the effects of *F. nucleatum* on OSCC pathogenesis and response to immunotherapy.

#### 4.2.3 Enhancement of antitumor immunity

Intratumoral microbes can retard cancer development by augmenting antitumor immunity. Gram-negative bacteria were detected in the cytoplasm of osteosarcoma cells and tumor-associated macrophages (TAMs) (74). Osteosarcoma tissues from patients with local diseases were enriched in Gram-negative bacteria compared with those from patients with metastatic status. Moreover, the amount of antitumor M1 macrophages was also increased in osteosarcoma patients with local diseases. It was likely that the abundance of Gram-negative bacteria was associated with the expansion of M1-polarized macrophages. This study provided new insight into the mechanisms underlying osteosarcoma progression as well as therapeutic approaches against cancer. The possible functional linkage between intratumor microbiota and macrophage-mediated immune surveillance needs to be explored in further studies. Reportedly, the TLR4 signaling coordinated macrophage polarization towards M1 phenotype (75). It is therefore questioned whether the TLR4 signaling pathway mediates the effect of intratumor bacteria on macrophage activation in osteosarcoma. Soft tissue sarcoma harbored bacterial (e.g., Bacteroidetes, Firmicutes and Proteobacteria) and viral species (51). *Respirovirus* was strikingly enriched in patients without metastases compared with those with metastases. The abundance of intratumoral viruses was positively associated with natural killer (NK) cell infiltration. NK cells with an antiviral phenotype were related to improved clinical outcomes in patients with soft tissue sarcoma, underscoring an interrelation between the intratumoral virome, NK cell infiltration and favorable patient outcomes. However, the role and clinical significance of intratumoral microbiota in soft tissue sarcoma warrant more detailed investigation.

Increasing evidence indicates that tumor-associated microbiota functions as the focal point of local immune activation. Intratumoral inoculation of *Neospora caninum* induced melanoma cell death and noticeably suppressed tumor growth in mice (52). Mechanistic investigation demonstrated that *N. caninum* administration enhanced the production of Th1 cytokines including interferon-γ (IFN-γ), IL-2, IL-10, IL-12, programmed death-ligand 1 (PD-L1) and tumor necrosis factor-α (TNF-α) in the TME of melanoma-bearing mice, resulting in extensive tumor cell death. The infiltration levels of CD8^+^ T cells and macrophages were increased in *N. caninum*-treated mice*. N. caninum* also reversed intestinal microbiota dysbiosis by elevating the relative abundance of *Adlercreutzia*, *Lachnospiraceae*, *Lactobacillus* and *Prevotellaceae*. It seemed that *N. caninum* exerted a direct tumorolytic effect, but the death pathway of cancer cells involved remains to be ascertained in future studies. It is also intriguing whether the role of *N. caninum* in intensifying antitumor immunity can be partially ascribed to its effects on gut microbiota. Albeit these unsolved questions, the efficacy, safety, stability and facile culture properties of *N. caninum* make it an appropriate biopharmaceutical drug for clinical cancer treatment. Tumor microbiota diversity and three genera (*Pseudoxanthomonas*, *Saccharopolyspora* and *Streptomyces*) were correlated with CD8^+^ T cell densities in PDAC, hinting that tumor microbiota might induce antitumor immunity through attraction and activation of CD8^+^ T cells (53). Human-to-mice faecal microbiota transplant (FMT) experiments showed that gut microbiota could modify tumor microbiota by directly migrating into PDAC tissues or altering intratumoral microbial composition. Importantly, FMT from long-term survival (LTS) donors could restrict PDAC growth in recipient mice. Antibiotic administration or CD8^+^ T cell depletion attenuated the antitumor effect induced by LTS FMT. Collectively, gut microbiota could translocate to pancreatic tumors and regulate tumor progression by manipulating tumor microbiome and shaping host immunity. More studies are required to illuminate how gut microbiota alters tumor microbiota and induces immune activation. Modulation of tumor-associated microbiota could be an effective therapeutic strategy for PDAC intervention. The efficacy of antitumoral microbiota therapies in PDAC must be validated in clinical studies.

#### 4.2.4 Suppression of antitumor immune cell function

In contrast, some intratumoral microorganisms exert an inhibitory effect on antitumor immune cells. A retrospective cohort study including 802 patients with nasopharyngeal carcinoma (NPC) showed that *Corynebacterium* and *Staphylococcus* dominated the composition of tumor microbiota (76). The intratumoral bacterial load inversely correlated with disease-free survival (DFS), distant MFS and OS. These observations implied that intratumoral bacterial load was a potential prognostic indicator in NPC. The bacterial load had a negative relationship with T lymphocyte infiltration, suggesting that NPC microbiota fostered tumor progression by dampening antitumor immunity. The detailed mechanisms underlying this association deserve in-depth investigation. *Anabaena* sp. K119 and *uncultured bacterium* were the most abundant microbes in lung adenocarcinoma (LUAD), while *Pseudomonas putida* str. KT2440 and *Thermostaphylospora chromogena* were enriched in lung squamous cell carcinoma (LUSC) (54). These four bacteria had a negative relationship with CD8^+^ T cells and macrophages and a positive association with monocytes and neutrophils. The high abundance of *P. putida* str. KT2440 in LUSC was linked with reduced infiltration levels of both activated dendritic cells (DCs) and naïve B cells. *T. chromogena* was connected with reduced infiltration of naïve B cells, mast cells, resting NK cells and CD4^+^ T cells. The decreased amount of *E. coli* str. K-12 substr. W3110 and the increased amount of *Staphylococcus aureus* in LUAD were significantly correlated with patient survival. *E. coli* str. K-12 substr. W3110 might display a pro-tumor property. On the contrary, *S. aureus* seemed to function like a tumor suppressor. However, ongoing studies are still needed to reveal the role of dysregulated microbes in the pathogenesis of lung cancer.

Intratumoral microbiota was highly organized in the microniches with epithelial and immune cell functions across human tumors including CRC and OSCC (55). Intratumoral microbiota enhanced the secretion of specific chemokines (e.g., C-C motif chemokine ligand 2 (CCL2), CCL4, CXCL1 and CXCL10) and interleukins (e.g., IL-1β, IL-6 and IL-10) into the surrounding milieu, leading to T cell exclusion and tumor growth. Furthermore, CRC-derived *F. nucleatum* recruited myeloid cells to initiate an inflammatory response through the Janus kinase (JAK)/STAT signaling pathway and promoted transcriptional alterations in CRC epithelial cells that fostered invasion to the surrounding environment. Collectively, intratumor microbiota formed an essential component of the TME that could influence the biology of various cellular compartments, contributing to the suppression of antitumor immunity and migration of cancer epithelial cells. Noguti et al. (56) revealed that the high abundance of *Eubacteria rectale/Roseburia* in colon cancer tissues was correlated with a decreased level of CD8^+^ T cells and an increased risk of tumor recurrence. The intratumoral microbiota might promote colon cancer progression by limiting antitumor immunity. More clinical studies are required to corroborate these results. The causal link between *E. rectale/Roseburia* and colon cancer development is worthy of future investigation. Concerted research efforts should be encouraged to better understand the role of specific microorganisms in modulating antitumor immune responses. Intratumoral *Methylobacterium* strikingly correlated with poor prognosis in GC patients (57). It showed a negative relationship with the level of transforming growth factor-β (TGF-β) as well as the frequency of CD8^+^ tissue-resident memory T (TRM) cells within the TME. The *in vivo* experiment verified that *Methylobacterium* could reduce TGF-β expression and CD8^+^ TRM cells in gastric tumor tissues. Collectively, intratumoral *Methylobacterium* functioned as a driver of gastric carcinogenesis. The underlying mechanism through which *Methylobacterium* induces the exhaustion of CD8^+^ TRM cells during GC development needs to be revealed.

The microbial abundance was significantly increased in human PDAC tissues compared with normal pancreas (7). Intra-pancreatic microbiota in PDAC-bearing patients was enriched in bacteria belonging to the phyla Actinobacteria, Bacteroidetes, Firmicutes and Proteobacteria and the genera *Elizabethkingia* and *Pseudomonas*. *B. pseudolongum* was the most abundant *Bifidobacterium* species in KC mice, which developed spontaneous pancreatic tumors. Oral antibiotic administration reduced the abundance of the pancreatic microbiota and suppressed pancreatic carcinogenesis. Therefore, the gut and PDAC microbiome facilitated cancer progression, raising the possibility that regulation of gut microbiota via probiotics could reduce PDAC risk. Oppositely, microbial ablation diminished the intratumoral infiltration of myeloid-derived suppressor cells (MDSCs), enhanced T cell infiltration and skewed macrophages polarization towards a tumor-suppressive M1 phenotype. Depletion of gut microbiota also promoted Th1 polarization of CD4^+^ T cells and potentiated the cytotoxic activity of CD8^+^ T cells, as shown by high expression levels of IFN-γ, T-bet and TNF-α. In addition, microbial ablation significantly elevated expression of programmed cell death protein-1 (PD-1) on effector T cells, implying that oral antibiotics combined with checkpoint-centered immunotherapy may be a promising therapeutic option for PDAC treatment. Conversely, re-population using faeces derived from PDAC-bearing KC mice or *B. pseudolongum* supplementation supported pancreatic oncogenesis in germ-free KC mice, however, this tumor-promoting effect was abolished in the absence of the TLR signaling. Further study indicated that cell free extracts from *B. pseudolongum* could reprogram TAMs to increase the levels of tolerogenic cytokines (e.g., IL-10) by activating the TLR signaling cascade. It was previously reported that macrophage polarization affected effector T cell function in PDAC (77). Consistently, macrophages entrained by cell free extracts from *B. pseudolongum* resulted in the inhibition of T cell immunity. Lipopolysaccharides and flagellins derived from intra-pancreatic bacteria (e.g., Proteobacteria) could be potential TLR activators, which remains to be verified in the future. PDAC microbiota might play a critical role in mediating immunosuppression by modifying the cytokine milieu. The pancreatic duct might act as the conduit for bacterial translocation from the intestinal tract to the pancreas. Successive research efforts are necessary to characterize the mechanisms regulating the migration of specific intestinal bacteria. Altogether, manipulation of PDAC microbiota may be helpful in enhancing the efficacy of cancer immunotherapy.

### 4.3 Modulation of cancer metabolic pathways

**Figure 4:**
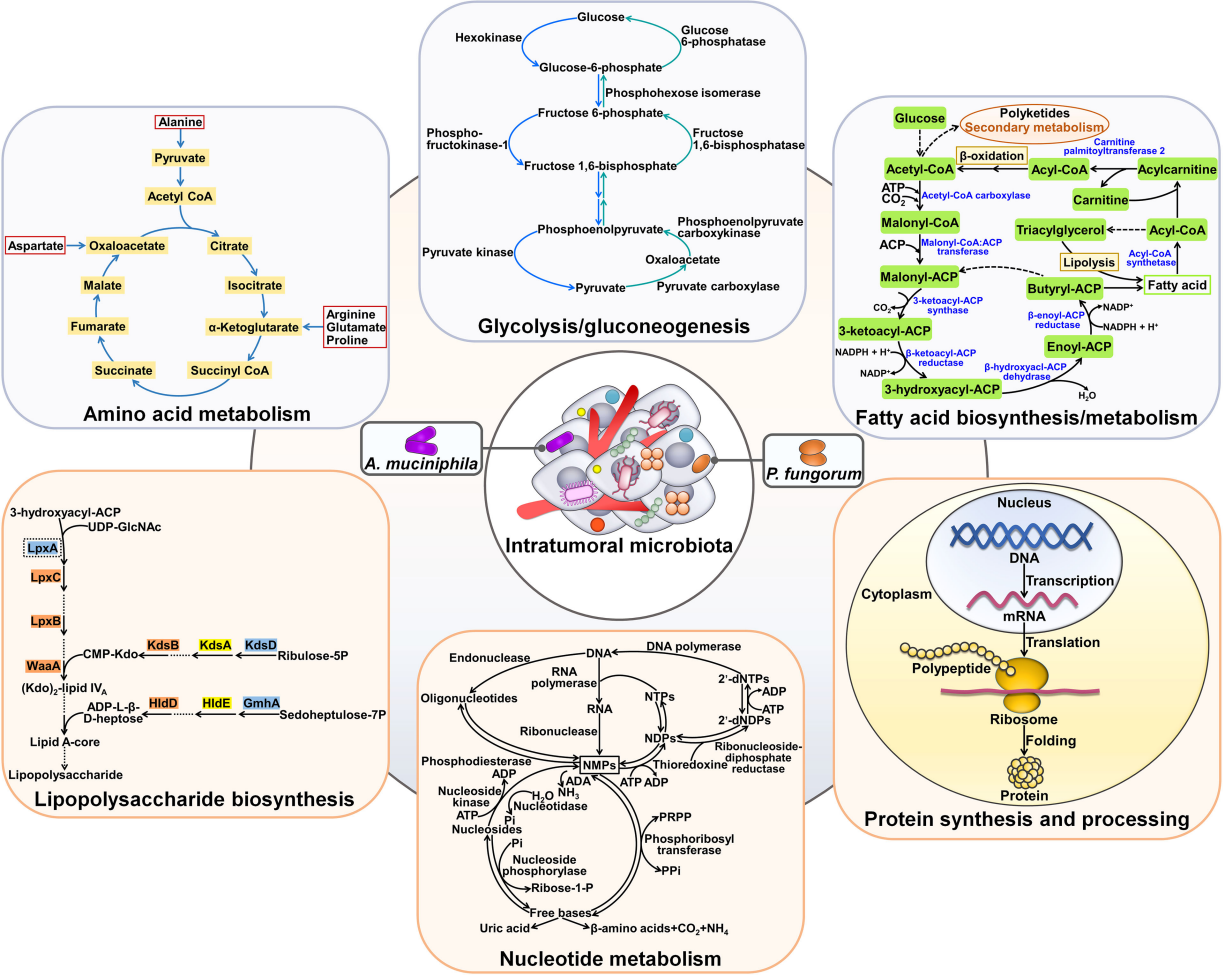
Effects of intratumoral microbiota on cancer metabolism. Tumor-resident microbes including *A. muciniphila* and *P. fungorum* can regulate amino acid metabolism. Moreover, intratumoral bacteria (e.g., *A. muciniphila*) affect glycolysis/gluconeogenesis and fatty acid biosynthesis and metabolism. In addition, intratumor microbial communities exert regulatory effects on lipopolysaccharide biosynthesis, nucleotide metabolism and protein synthesis and processing.

Cancer metabolism is key and intimately linked to cancer progression. In recent years, the role of intratumoral microbiota in reprogramming of cancer metabolism has been increasingly recognized (**Table 2**). Bacillales, Burkholderiales, Clostridiales, Pseudomonadales, Sphingomonadales and Xanthomonadales were abundant in ICC tissues (58). *Klebsiella pneumoniae*, *Paraburkholderia fungorum*, *Pseudomonas azotoformans* and *Staphylococcus capitis* were verified to be present in ICC tissues. Intratumoral bacteria mainly resided in malignant cells of cancerous tissues, while they also localized to T cells, hinting that intratumor microbiota might be involved in tumor immunity. The amount of *P. fungorum* was noticeably higher in paracancerous tissues and had an inverse association with the carbohydrate antigen 199 (CA199) level, alluding to the potential antitumor potency of this bacterium. As expected, *P. fungorum* could repress the growth of human cholangiocarcinoma cells *in vivo* by regulating the metabolism of alanine, aspartate and glutamate (**Figure 4**). The hepatic-intestinal circulation may contribute to the crosstalk between intestinal bacteria and intratumoral microbes. The impact of intratumoral microbes on cancer metabolism should be an important subject for future investigation. *Akkermansia muciniphila* exhibited an inhibitory activity against lung cancer growth *in vivo* (59). This intestinal commensal bacterium could translocate to lung cancer tissues via systemic circulation. *A. muciniphila* altered the intratumoral metabolic pathways including amino acid metabolism, glycolysis/gluconeogenesis and fatty acid biosynthesis. Tumor-associated *A. muciniphila* also modified the composition of intratumor microbiota by increasing the abundance of Acidobacteriae, *Akkermansia*, *Bacteroides*, *Bifidobacterium*, Gammaproteobacteria, *Lactobacillus*, Sphingomonadales and *Staphylococcus*. *A. muciniphila*-influenced symbiotic microecology showed an association with the metabolic network in tumor tissues. Continual efforts should be made to clarify intratumoral microbe-mediated complicated cancer metabolic reprogramming. The specific intracellular microorganisms that are involved in the antitumor action of *A. muciniphila* should be identified.

Bacterial communities were present in HCC cells and immune cells (78). Microbial diversity and richness were dramatically increased in HCC tissues compared with paracancerous tissues. The levels of commensal *Enterobacteriaceae*, *Fusobacterium* and *Neisseria* were higher in HCC tissues than in paracancerous tissues, whereas *Agathobacter*, *Chryseobacterium*, *Dietzia*, *Faecalibacterium*, *Hydrogenophaga*, *Megamonas* and *Pseudomonas* exhibited the opposite trend. It was reported that HCC-enriched *Enterobacteriaceae* utilized inflammatory byproducts within the microniches as energy sources, which eventually led to inflammation (79, 80). Likewise, the proinflammatory property of *Fusobacterium* was also documented (81). Since inflammation has been acknowledged as a risk factor for carcinogenesis, these bacteria may play a role in HCC formation (82). The high infiltration of pro-tumorigenic bacteria (e.g., *Neisseria*) and the reduced abundance of the tumor-suppressive bacteria (e.g., *Pseudomonas*) potentially propel HCC development (83, 84). Importantly, intratumoral microbes could alter cancer metabolic pathways. Fatty acid and lipopolysaccharide biosynthesis, and bacteria and disease pathways were remarkably enriched in HCC tissues. The resultant products of microbial metabolic activities may be a key source of fatty acids and lipids for cancer cells, thereby facilitating HCC cell proliferation and invasion. Intestinal permeability may be an explanation for bacterial translocation into HCC, calling for further verification. The mechanisms of action of intratumoral microbes in HCC carcinogenesis should be further explored.

Tumor microbiota communities were markedly different between PDAC patients with short-term survival (STS) and those with LTS (53). The STS tumors showed a predominance of Bacteroidea and Clostridia, while PDAC LTS cases were dominated by Alphaproteobacteria, Flavobacteria and Sphingobacteria. Actinobacteria (*Saccharopolyspora* and *Streptomyces*) and Proteobacteria (*Pseudoxanthomonas*) were more abundant in LTS patients versus STS patients. The heterogeneous tumor microbiota signatures contributed to different enrichment of metabolic functional pathways between STS and LTS patients. For instance, the STS cases were enriched in the pathways related to nucleotide metabolism, replication and repair, protein synthesis and processing, while the LTS cases demonstrated enrichment in the pathways associated with metabolism of amino acid, lipids and polyketides. The divergence in metabolic pathways may be an explanation for different clinical outcomes in PDAC patients, which necessitates additional research.

### 4.4 Regulation of cellular signaling pathways

**Figure 5:**
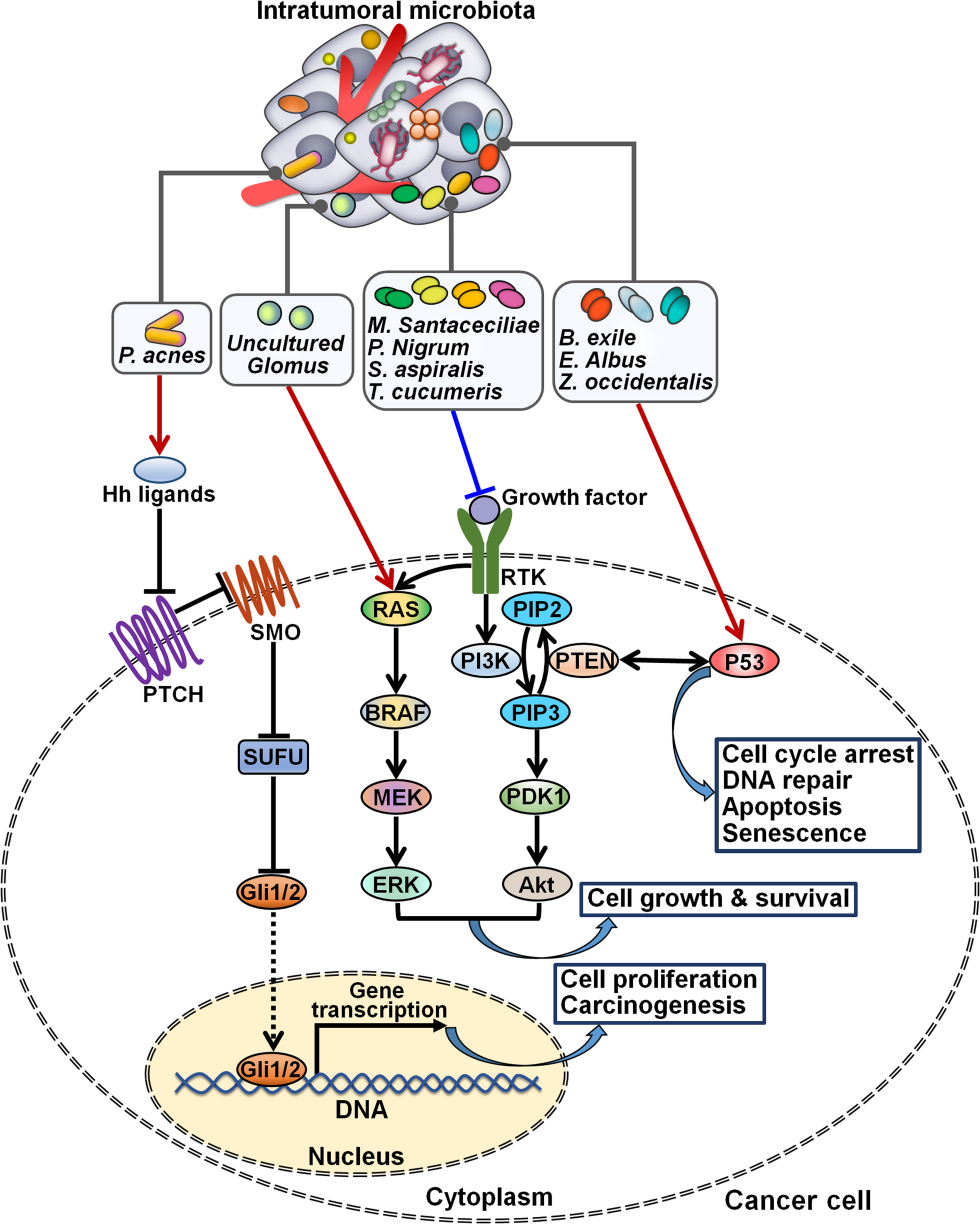
Regulation of intracellular signaling pathways by intratumoral microbiota. Intratumoral microbiota regulates the occurrence and development of cancer by coordinating diverse signal transduction cascades. The proinflammatory *P. acnes* induces carcinogenesis by activating the Hh signaling cascade. *Uncultured Glomus* is associated with the enhancement of BRAF kinase activity and the activation of the MAPK signaling cascade, while *M. santaceciliae*, *P. nigrum*, *S. aspiralis* and *T. cucumeris* are capable of inhibiting the PI3K/Akt pathway. *B exile*, *E albus* and *Z. occidentalis* act to motivate the p53 signaling cascade. Hh, Hedgehog; PTCH, patched homolog; SMO, smoothened homolog; SUFU, suppressor of fused; Gli, glioma-associated oncogene; RAS, rat sarcoma viral oncogene homolog; BRAF, B-Raf proto-oncogene serine/threonine kinase; MEK, mitogen-activated extracellular signal-regulated kinase; ERK, extracellular signal-regulated kinase; RTK, receptor tyrosine kinase; PI3K, phosphatidylinositol 3-kinase; PIP2, phosphatidylinositol-4,5-bisphosphate; PTEN, phosphatase and tensin homolog; PIP3, phosphatidylinositol-3,4,5-triphosphate; PDK1, 3-phosphoinositide-dependent kinase 1; Akt, protein kinase B.

Intratumoral microbiota functions as key players in cancer-associated signaling cascades. The pathogens *Acinetobacter*, Actinomycetales, *Ochrobacterium*, *Pseudomonas* and *Streptococcus* were enriched in epithelial ovarian cancer (EOC) tissues (60). Intratumoral inoculation of *Propionibacterium acnes* propelled tumor growth and shortened the survival rate of EOC-bearing mice. The *P. acnes* strain could enhanced the expression of TNF-α and IL-1β, suggesting the proinflammatory potency of this bacterium. These two proinflammatory cytokines that were excreted by necrotic tumor cells could enhance Hedgehog (Hh) transcription (85). As expected, the Hh signaling pathway was abnormally motivated in *P. acnes*-treated mice, as evidenced by increased expression of glioma-associated oncogene 1 (Gli1), Gli2, patched homolog 1 (PTCH1), sonic hedgehog homolog (Shh) and smoothened homolog (SMO). Conversely, blockade of the Hh signaling prevented EOC progression caused by *P. acnes*. The proinflammatory *P. acnes* facilitated EOC pathogenesis by activating the Hh signaling cascade (**Figure 5**). Complementary *in vitro* and *in vivo* studies are warranted to delve into how intratumoral microbes interact with intracellular signaling pathways, providing important clues for improving cancer management.

The fungal species including *Metarhizium acridum CQMa 102*, *Phaffia rhodozyma* and *S. cerevisiae YJM1338* were enriched in tumor tissues of patients with papillary thyroid carcinoma (PTC) (61). Three PTC subtypes, Classical (CPTC), Follicular Variant (FVPTC) and Tall Cell (TCPTC), showed a similar microbial composition and richness. For instance, *Botrytis cinerea*, *Pichia cephalocereana* and *Trematosphaeria pertusa* were overabundant in these subtypes. *Rhizopus arrhizus* and *uncultured Uromyces* were underabundant in both CPTC and TCPTC. The level of *Chaetomium globosum CBS 148.51* was related to increasing pathologic stage in PTC patients. *C. Albicans*, *Eremascus albus* and *Thanatephorus cucumeris* were positively associated with a higher pathologic M stage, while *Spiromyces aspiralis*, *uncultured Cryptomycota* and *Wickerhamiella pararugosa* had a linkage with a higher pathological N stage. Further studies are required to characterize the influence of these fungi on PTC tumor metastasis. Importantly, the relative abundance of fungal microbes was related to PTC-specific oncogenic pathways. For instance, *Metschnikowia santaceciliae*, *Pacynthium nigrum*, *Spriromyces aspiralis* and *T. cucumeris* were associated with the downregulation of the phosphatidylinositol 3-kinase (PI3K)/protein kinase B (Akt) pathway in CPTC. *Brevicellicium exile*, *E. albus* and *Zoophthora occidentalis* correlated with the upregulation of the p53 signaling in TCPTC. *Uncultured Glomus* was linked with increased activity of BRAF kinase and the activation of the mitogen-activated protein kinase (MAPK) signaling in FVPTC. Additional investigation is needed to identify intracellular signaling pathways that mediate the tumor-promoting effects of intratumoral fungal species. The intricate signaling network underlying PTC development merits further study.

## 5 Impact of intratumoral microbiota on anticancer therapy

**Figure 6:**
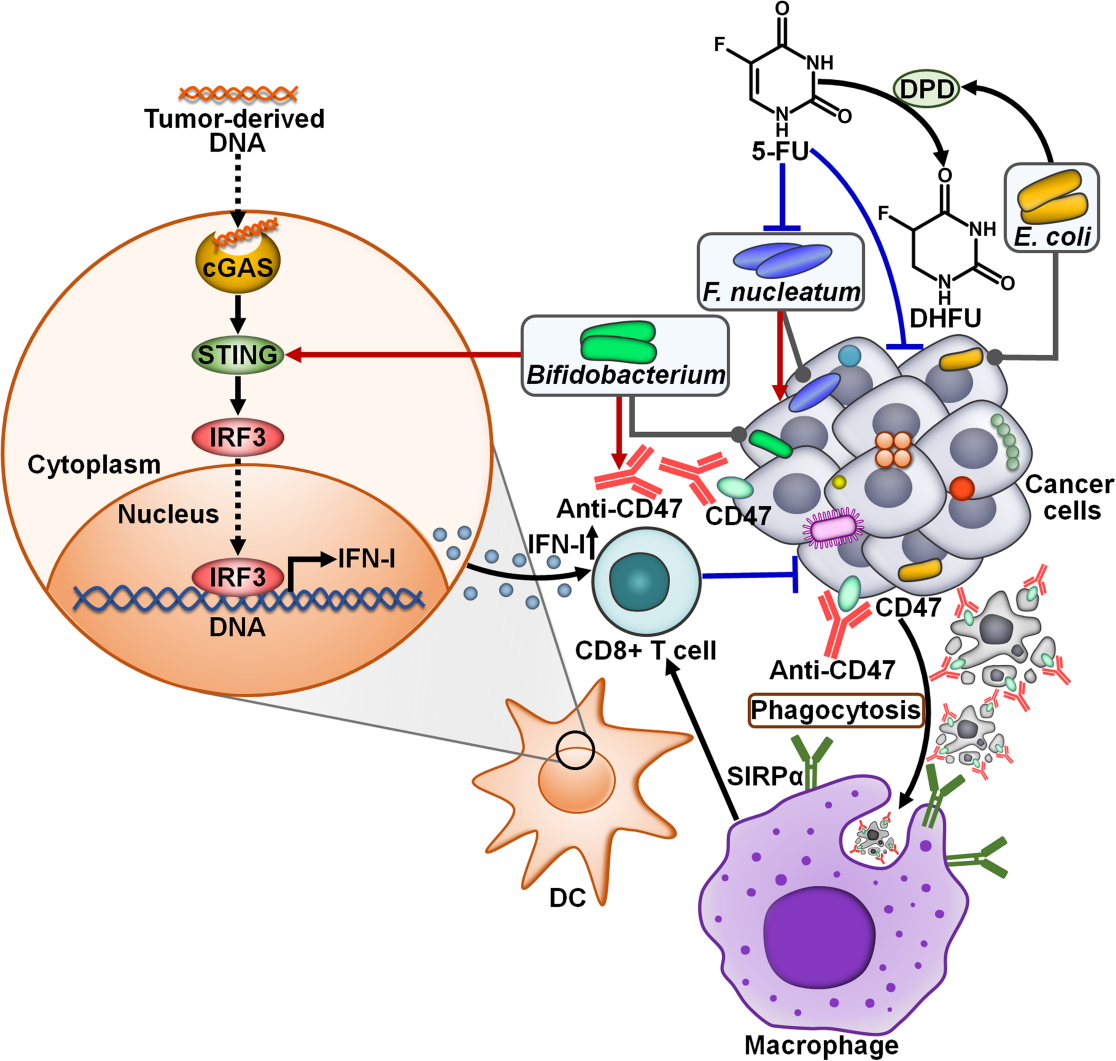
Impacts of intratumoral microbiota on the antitumor efficacy of cancer treatment. CD47, a surface membrane protein expressed by some cancer cells, binds to SIRPα on the surface of macrophages, contributing to impairment of phagocytic capacity. Blocking CD47 with anti-CD47 promotes phagocytosis of cancer cells by macrophages, which further initiates cytotoxic CD8^+^ T cell responses. *Bifidobacterium* can augment the antitumor effects of CD47-based therapy. In term of mechanisms, *Bifidobacterium* can actuate the STING signaling to enhance IFN-I production, resulting in the activation of CD8^+^ T cell-mediated immune responses. The intratumoral bacteria *E. coli* dampens the toxicity of 5-FU toward tumor-supportive *F. nucleatum* and cancer cells by transforming 5-FU into nontoxic DHFU. cGAS, cyclic guanosine monophosphate-adenosine monophosphate synthase; STING, stimulator of interferon genes; IRF3, interferon regulatory factor 3; IFN-I, type I interferon; DC, dendritic cell; 5-FU, 5-fluorouracil; DPD, dihydropyrimidine dehydrogenase; DHFU, dihydrofluorouracil; SIRPα, signal regulatory protein α.

There is a growing appreciation of the effect of intratumoral microbiota on the response to anticancer therapies (**Table 2**). *F. nucleatum*, a predominant bacterium within CRC microbiota, was identified as a tumor-supportive bacterial species that was correlated with inferior patient prognosis (86). The first-line CRC chemotherapeutic drug, 5-fluorouracil (5-FU), exhibited an antagonistic activity against *F. nucleatum* (62). The intratumoral bacteria *E. coli* was resistant to 5-FU and could attenuate 5-FU toxicity toward *F. nucleatum* and human CRC epithelial cells (**Figure 6**). Mechanistically, *E. coli* contained a functional homolog of human dihydropyrimidine dehydrogenase (DPD) that converted 5-FU to nontoxic dihydrofluorouracil (DHFU). Thus, intratumoral microbiota had a significant impact on cancer cell sensitivity to chemotherapy. CRC patient-derived *ex vivo* tumor microbiota underwent community disruption upon exposure to 5-FU, characterized by an expansion of 5-FU-resistant bacteria (e.g., *E. coli*). The altered microbiota caused the depletion of 5-FU and thus blunted local chemotherapeutic efficacy. An improved understanding of the effect of intratumoral microbes on patient response to chemotherapy would assist in stratifying patients for combined treatment of chemotherapy with targeted antimicrobials. Further investigation into the detailed mechanisms behind intratumoral bacteria-mediated chemotherapeutic resistance are warranted.

Two mice models (Jax and Tac) had different gut microbiota profiles that led to distinct immune signatures (65). CRC-bearing Jax mice responded to anti-CD47 immunotherapy, while tumor-bearing Tac mice exhibited low responsiveness to CD47 blockade (63). Cohousing of tumor-bearing Jax mice with Tac mice restored the response of mice nonresponders (Tac mice) to CD47-based immunotherapy. Intriguingly, intratumoral inoculation of the antibiotic cocktail reduced the efficacy of anti-CD47 treatment in mice responders, hinting that intratumoral microbiota affected the efficacy of anti-CD47 immunotherapy. It was proposed that hypoxic environment within tumor tissues was beneficial for the colonization and growth of anaerobic commensals. Systemic or intratumoral administration of the *Bifidobacterium* cocktail (*B. bifidum*, *B. breve*, *B. lactis* and *B. longum*) rescued the capability of tumor inhibition by CD47 blockade in mice nonresponders. Intratumoral administration of the antibiotic cocktail blunted the therapeutic potency of *Bifidobacterium*-facilitated CD47 blockade. It could be concluded that *Bifidobacterium* had tumor-targeting ability and was essential for anti-CD47-mediated tumor inhibition effect. Moreover, specific inhibition of type I IFN (IFN-I) signaling in DCs within the TME dampened the antitumor efficacy of CD47-based therapy in mice nonresponders administrated with *Bifidobacterium*. The expression level of IFN-β was higher in tumor DCs derived from mice nonresponders treated with *Bifidobacterium* and CD47 blockade than those from mice only administrated with anti-CD47. The antibiotic cocktail-mediated removal of *Bifidobacterium* inside the TME attenuated IFN-β expression in tumor DCs, and also inhibited their cross-priming ability. *Bifidobacterium* administration failed to rescue the antitumor efficacy of CD47 blockade in T cell-deficient mice as well as in stimulator of interferon genes (STING)-knockout mice. Specific depletion of STING inside DCs also blocked the antitumor function of *Bifidobacterium*. Oppositely, the STING agonist enhanced the antitumor capacity of CD47 blockade in nonresponding mice. These results suggested that *Bifidobacterium* strengthened the antitumor effects of CD47-based therapy in a STING signaling- and T cell-dependent manner. It is likely that other anaerobic commensal microbes also have tumor-targeting potential and induce antitumor immunity. It is imperative to investigate whether these microorganisms accumulate in the TME and influence the antitumor effect of immunotherapies. Manipulation of the microbiota inside TME may represent an effective strategy to improve immunotherapeutic effectiveness. However, the pathogenicity of tumor-targeting bacteria must be adequately determined. *Bifidobacterium* is an anaerobic commensal bacterium with low toxicity and low possibility of residing in normal tissues, which make it a suitable tumor-targeting bacterium for clinical cancer intervention. Active bacteria can generate secondary metabolites to activate the STING signaling inside DCs. The mechanisms through which *Bifidobacterium* motivates the STING pathway remain to be determined. Special attention should be paid to the role of *Bifidobacterium* in antitumor immunity.

The core purpose of cancer immunotherapy is the persistent activation of tumor-specific T cells (87). Immune checkpoint-directed therapy can induce durable antitumor immune responses in cancer and has attracted increasing scientific and clinical interests in recent years. Intratumoral microbiota can affect the anticancer effect of immune checkpoint therapy. For instance, depletion of PDAC microbiota enhanced the efficacy of PD-1-targeted immunotherapy by increasing PD-1 expression (7). Introduction of *Megasphaera* into the breast cancer microenvironment yielded a better inhibitory effect on tumor growth when combined with anti-PD-1 therapy *in vivo* (64). Commensal *Bifidobacterium* promoted CD8^+^ T cell accumulation in the TME (65). The combination of *Bifidobacterium* administration and anti-PD-L1 treatment exerted a synergistic antitumor effect on melanoma. Short chain fatty acid (SCFA)-producing bacteria, including *Eubacterium*, *Lactobacillus* and *Streptococcus*, could improve the efficacy of anti-PD-1/PD-L1 treatment in gastrointestinal cancer (66). This effect might be attributed to the immunoregulatory function of SCFAs, which necessitates further study. In addition, *B. fragilis* elicited Th1 immune responses and favored the maturation of intratumoral DCs, which potentiated the therapeutic efficacy of cytotoxic T lymphocyte-associated protein 4 (CTLA-4) blockade in fibrosarcoma-bearing mice (67). Despite various studies indicating the relationship between intratumoral microbiota and immunotherapy effect, the underlying mechanisms remain equivocal. A deeper understanding of the influence of intratumoral microbiota on immunotherapy efficacy will provide a new direction for the clinical treatment of cancer.

## 6 Clinical implications of intratumoral microbiota in cancer

### 6.1 Cancer diagnosis

**Figure 7:**
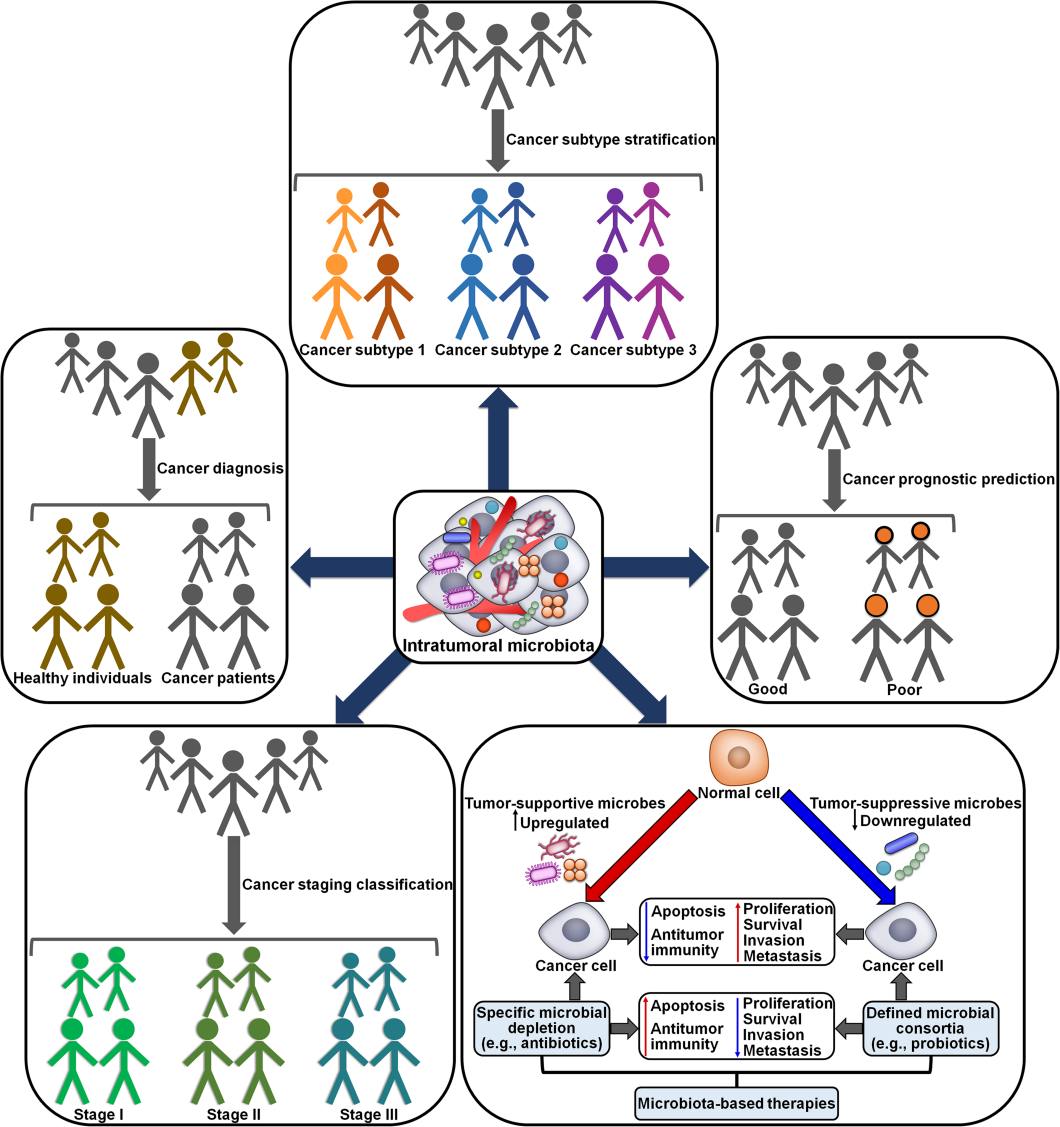
Potential clinical implications of intratumoral microbiota in cancer. Specific tumor-associated microbiota signatures are capable of discriminating cancer patients from healthy individuals. The microbial biomarkers also differentiate between and within subtypes and stages of cancers. Tumor-resident microbes may provide a valuable tool for cancer diagnosis, subtype stratification and staging classification. The abundance and diversity of intratumoral microbes are associated with clinical outcomes in cancer patients. Thus, intratumoral microbiota may have a promising role in cancer prognosis. Considering its important role in cancer pathogenesis, intratumoral microbiota may represent an attractive therapeutic target for cancer treatment. Microbiota modulation, such as depletion of tumor-supportive microbes and introduction of tumor-suppressive microbes, shows great potential in improving cancer treatment outcomes.

The momentous role of intratumoral microbiota in cancer development and tumor immunity highlights its potential clinical implications in oncology. The diagnostic values of intratumoral microbiota have been explored. Wang et al. (88) revealed that head and neck squamous cell carcinoma (HNSCC)-depleted Actinobacteria/Actinomycetales/*Actinomyces*, Firmicutes, Lactobacillales, *Rothia*, *Streptococcus* and Veillonellales had solid predictive ability in differentiating tumor tissues from normal tissues. This intratumoral microbiota signature was associated with the clinicopathological characteristics such as age, gender, tumor stage and neoplasm histological grade. A microbial signature consisting of HCC-depleted Acidobacteriae and Parcubacteria as well as HCC-enriched Bacilli, Gammaproteobacteria and Saccharimonadia exhibited great discriminative accuracy and performance in HCC prediction (44). HCC-enriched Actinobacteriota, Firmicutes, Gammaproteobacteria, Proteobacteria and Saccharimonadia correlated with clinicopathological characteristics of HCC patients including tumor volume, cirrhosis grading (inflammation activity) and histological severity. These microbes might contribute to the onset and development of HCC. Intratumoral microbial signatures may be a valuable resource for unearthing prospective biomarkers for cancer diagnosis (**Figure 7**). The potential utility of specific intratumoral microbes in clinical settings should be illuminated in the future. Further study on the association between specific microbes and clinicopathological features is required to support the aforementioned results.

### 6.2 Cancer subtype stratification

A microbial signature comprising Actinobacteria, Bacilli, Bacillales, Epsilonproteobacteria, Fusobacteria, *Fusobacteriaceae*, *Lactobacillaceae*, Negativicutes, Pasteurellales and *Streptococcaceae* could be a potential biomarker for subdividing different ESCA subtypes (ESCC and esophageal adenocarcinoma (EAD)) (89). ESCA-enriched Fusobacteriales positively correlated, while the microbial abundance of Lactobacillales inversely correlated with the tumor stage. Fusobacteria, present in multiple cancer types, promoted the establishment of a proinflammatory niche that supported cancer development (90, 91). Therapeutic manipulation of Fusobacteria could be a promising treatment option in cancer patients. High levels of *Lactobacillus*, Negativicutes and Proteobacteria reflected better prognosis, while the enrichment of Clostridiales and Fusobacteriales was associated with worse prognosis. Clostridia, Clostridiales, Fusobacteriia and Fusobacteriales might be independent prognostic biomarkers for ESCA. Orally derived *Fusobacterium* species including *Fusobacterium animalis*, *F. nucleatum*, *Fusobacterium polymorphum* and *Fusobacterium vincentii* were prevalent in tumor tissues of CRC patients (92). The *Fusobacterium* species were enriched in right-sided, microsatellite instability-high (MSI-H) and BRAF-mutant tumors. *Fusobacterium* might have prominent specificity for the inflamed CRC subtype. *F. animalis* was likely to correlate with disease progression and reduced survival in consensus molecular subtype 4 (CMS4) CRC patients, highlighting that intratumoral microbes might represent promising biomarkers for CRC subtype stratification and prognosis. GC tissues had a lower diversity of microbiota than adjacent nonmalignant tissues (93). MSI-H subtype might have a tendency toward an increased microbial diversity relative to other subtypes (diffuse and intestinal GC). The diversity of intratumoral microbiota may be a potential biomarker for the classification of GC subtypes. Further investigation of intratumoral microbial signatures will help to better understand key pathological processes and biological mechanisms during cancer pathogenesis, as well as facilitate the identification of new microbial biomarkers for cancer.

### 6.3 Cancer staging classification

The high levels of *Fusobacterium* and *Haemophilus* correlated with the T3/T4 stage and lymphatic metastasis in patients with oral cancer (94). Bacillales and *Gemella* were more abundant in T1/T2 stage patients and the non-lymphatic metastasis cases. These results demonstrated that intratumoral microbiota could be connected with oral cancer progression and metastasis. Future work should concentrate on delineating the characteristics of the microbiota in metastatic lymph nodes and the mechanisms through which intratumoral microbes affect lymphatic metastasis. *Acinetobacter*, *Pseudomonas*, *Ralstonia*, *Rhodococcus* and *Sphingomonas* were the dominant bacteria in PTC tissues (95). PTC patients with advanced lesions had a higher microbiota diversity than patients with mild lesions. The abundance of *Pseudomonas*, *Rhodococcus* and *Sphingomonas* was high in mild lesion cases. *Granulicatella* and *Streptococcus* were more abundant in patients with advanced lesions than those with mild lesions. The eight-genera microbiota signature that was composed of *g_norank_F_norank_o_Coriobacteriales*, *g_unclassified_o_Rhizobiales*, *Granulicatella*, *Haemophilus*, *Pseudomonas*, *Rhodococcus*, *Sphingomonas* and *Streptococcus* had the potential to discriminate PTC invasion status. Intratumoral microbes potentially interacted with thyroid hormones and autoimmune antibodies, which remodeled the TME, contributing to PTC invasion and progression. The mechanism by which tumor-resident microbiota affects PTC development and outcome necessitates substantial attention. The increased abundance of *Blumeria graminis f.* sp. *Hordei* was strikingly associated with early cancer pathological stages in HNSCC, while the decreased number of *Aspergillus flavus*, *Coccidioides immitis RS* and *Gaeumannomyces tritici R3-111a-1* correlated with the absence of perineural invasion (96). The high level of *Podospora anserina S mat^+^* and *S. cerevisiae EC1118* was linked with tumor neoplasm presence and advanced pathological N-stage. These altered microbes might give rise to cancer metastasis and progression. Moreover, *Inosperma fulvum*, *Phlyctochytrium arcticum* and *uncultured Cryptomycota* had a relationship with clinical characteristics of patients with HPV^+^ HNSCC. *Punctularia strigosozonata HHB-11173 SS5* was linked to the upregulation of oncogenic pathways and poorer patient prognosis. These fungal microbes might be clinically relevant to patient outcome, but their genuine roles in HNSCC development needs further study.

### 6.4 Cancer prognostic prediction

Pathogenic oral microbes, including Firmicutes, Fusobacteriia, Fusobacteriales, *Fusobacteriaceae* and *Fusobacterium*, exhibited great predictive efficacy in OSCC diagnosis and prognosis (97). These microorganisms correlated with clinical characteristics of OSCC, including histological grade and tumor stage. Intratumoral microorganisms could be used as diagnostic and prognostic biomarkers for OSCC. The association between oral microbiome and clinical parameters of OSCC necessitates further clinical studies. *H. parainfluenzae* might be negatively associated with the first-line treatment outcomes in NSCLC patients (98). *Staphylococcus crista* and *Staphylococcus haemolyticus* were related to longer progression-free survival (PFS). *Serratia marcescens* was associated with better OS while *Corynebacterium jergeri* and *H. parainfluenzae* were associated with poorer OS. It is still obscure how intratumoral microbiota influences the treatment and prognosis of lung cancer. A microbiome analysis on biopsy samples from different sites of patients with distinct primary tumors (breast, CRC and lung) showed that *Acinetobacter*, *Burkholderia*, *Corynebacterium*, *Cutibacterium*, *Flavobacterium*, *Pelomonas*, *Rheinheimera*, *Sphingobium*, *Staphylococcus* and *Streptococcus* were the ten most represented genera in metastatic tumor tissues (99). The richness of intratumoral microbiota was significantly related to OS and PFS in cancer patients. Further experiments to determine the clinical value of intratumoral microbiota are required. The relative abundance of intratumoral *Pseudomonas* was significantly higher in PLC patients with LTS than in those with STS (17). The level of *Pseudomonas* was positively correlated with clinical prognosis in PLC patients. This bacterium might represent a prognostic biomarker for PLC. Tumor microbial diversity was positively correlated with OS in patients with resected PDAC (53). The increased number of *Pseudoxanthomonas*, *Saccharopolyspora* and *Streptomyces* was associated with better clinical outcomes in PDAC patients. Another study showed that the composition and diversity of intratumoral microbiota were apparently distinct between long-term and short-term PDAC survivors (64). The high abundance of *Megasphaera* and *Sphingomonas* positively correlated with the prolonged OS. The high level of opportunistic pathogen *Clostridium* was related to shortened survival time. These dysregulated microbes might be promising prognostic biomarkers for PDAC. The high load of intratumoral *F. nucleatum* was markedly related to better OS, DFS and MFS in patients with anal squamous cell carcinoma (ASCC) (100). In addition, the association between intratumoral fungi and patient survival and therapy response in different cancer types was also documented (9). The oncogenic fungus, *M. globosa*, was correlated with shorter OS in breast cancer patients (49). The *Phaeosphaeria* genus was dramatically linked with shorter PFS in patients with ovarian cancer. The high amount of *Cladosporium* might be negatively associated with immunotherapy response in patients with metastatic melanoma. Thus, intratumoral fungi hold the promise as potential biomarkers and therapeutic targets in cancer. The clinical implication of intratumoral fungi remains to be detected in larger cohorts. Given a paucity of publicly-available fungal genomes, a great deal of investigative work is required to better understand the fungal functional repertoires in cancer.

### 6.5 Anticancer therapy

In light of recent advances in the field of intratumor microbiome, microbiota-based therapies are considered to hold great application potential (**Figure 7**). Many clinical trials exploring the therapeutic benefits of wild-type/modified microorganisms in cancer patients are now underway (**Table 3**). A randomized, placebo-controlled, phase I dose-escalation trial in 26 patients with advanced PC indicated the safety, immunogenicity and tolerability of an oral T cell vaccine VXM01, which was composed of live attenuated *Salmonella typhi* carrying a eukaryotic expression plasmid encoding vascular endothelial growth factor receptor 2 (VEGFR2) (101). Another phase I clinical trial demonstrated that the VXM01 vaccine was efficacious in inducing VEGFR2-specific effector T cell responses and attenuating tumor perfusion in 30 patients with advanced PC (102).

**Table 3:** Overview of published clinical trials of antitumor microbial therapies.

Microbial species	Drug	Type	Route of administration	Cancer type	Type of study	Number of subjects	Outcome	Reference
*Salmonella typhi*	VXM01	Live attenuated	Oral	Advanced pancreatic cancer	Randomized, placebo-controlled, phase I dose-escalation trial	26	Induce vaccine specific T cell responses	(101)
*Salmonella typhi*	VXM01	Live attenuated	Oral	Advanced pancreatic cancer	Randomized, dose-escalation phase I clinical trial	30	Induce vaccine specific effector T cell responses and reduce tumor perfusion	(102)
*Listeria monocytogenes*	CRS-207	Live attenuated	Intravenous	Malignant pleural mesothelioma	Multicenter, open-label phase Ib study	35	Induce antitumor immunity and objective tumor responses	(103)
*Listeria monocytogenes*	CRS-207	Live attenuated	Intravenous	Pancreatic cancer	Multicenter, randomized, phase II trial	90	Induce mesothelin-specific CD8 T cell responses and prolong survival of patients	(104)
*Listeria monocytogenes*	CRS-207	Live attenuated	Intravenous	Pancreatic cancer	Randomized, controlled phase IIb clinical trial	303	Provide comparable survival benefits with standard chemotherapy	(105)
*Listeria monocytogenes*	ADXS11-001	Live attenuated	Intravenous	HPV-associated oropharyngeal cancer	Phase II “window of opportunity” trial	8	Induce T cell responses and correlate with increased serum CCL22 level	(106)
*Listeria monocytogenes*	ADXS11-001	Live attenuated	Intravenous	Anal cancer	Phase I trial	9	Delay disease progression	(107)
*Listeria monocytogenes*	ADXS31-142	Live attenuated	Intravenous	Metastatic castration-resistant prostate cancer	Open-label phase I/II KEYNOTE-046 study	50	Improve overall survival	(108)
*Listeria monocytogenes*	ADXS31-164	Live attenuated	Intravenous	HER2/neu-positive cancers	Phase I trial	–	Induce strong T cell immune responses, inhibit tumor growth and extend overall survival	(109)
*Clostridium novyi*	*C. novyi*-NT	Live attenuated	Intratumoral	Treatment-refractory advanced solid tumors	Open-label, multicenter, phase I study	24	Induce a transient systemic cytokine response, promote tumor-specific T cell responses and reduce tumor size	(110)

CRS-207 is a live attenuated *Listeria monocytogenes* expressing mesothelin (103). In a multicenter, open-label phase Ib study, CRS-207 infusion in combined with chemotherapy decreased tumor size in 35 patients with malignant pleural mesothelioma (MPM). CRS-207 treatment induced marked changes in the local TME, as evidenced by elevated infiltration levels of T cells, DCs and NK cells, an increased ratio of CD8^+^ T cells to regulatory T cells (Tregs), and the transformation of M2-like macrophages into M1-like macrophages (103). Accordingly, CRS-207 combined with chemotherapy resulted in enhanced antitumor immunity and induced objective tumor responses in MPM patients. A multicenter, randomized, phase II trial involving 90 PC patients indicated that administration of CRS-207 plus cyclophosphamide (Cy)/the cancer vaccine GVAX exhibited favorable safety profile (104). The combined treatment promoted mesothelin-specific CD8 T cell responses and prolonged survival of PC patients. However, a randomized, controlled phase IIb clinical trial in patients with advanced PC showed that CRS-207 in combination with Cy/GVAX did not improve OS over standard chemotherapy (105). The engineered *Listeria* strain ADXS11-001, which expresses the HPV16-E7 fusion protein to target HPV-positive tumors, could activate antigen-specific T cell responses and lead to the increased serum level of CCL22 in patients with HPV-associated oropharyngeal cancer (106). The combination of ADXS11-001 with standard chemoradiation was proven to be well tolerable in patients with anal cancer (107). All nine patients showed complete clinical response, and eight patients (89%) exhibited no evidence of disease at a median follow-up of 42 months. ADXS31-142 is an attenuated *L. monocytogenes*-based immunotherapy targeting prostate-specific antigen (PSA) (108). An open-label phase I/II KEYNOTE-046 study including 50 patients with metastatic castration-resistant prostate cancer (mCRPC) preliminarily verified the safety and tolerability of ADXS31-142 combined with pembrolizumab. This combination treatment might improve OS in mCRPC patients with visceral metastasis, which warrants additional evaluation in future studies. ADXS31-164, a highly attenuated, recombinant *L. monocytogenes* expressing a chimeric human HER2/neu fusion protein, induced robust T cell immune responses, retarded tumor growth and extended OS in animal models (109, 111). ADXS31-164 might have significant translational relevance for patients with HER2/neu-positive cancers. An open-label, multicenter, phase I study demonstrated that a single intratumoral inoculation of nontoxic *Clostridium novyi* (*C. novyi*-NT) reduced the size of the injected tumor in nine patients with injectable, treatment-refractory solid tumors (41%) (110). *C. novyi*-NT administration induced a temporary systemic cytokine response and potentiated tumor-specific T cell responses. However, *C. novyi*-NT treatment might raise safety concerns including limb abscess, pathological fracture, rash and respiratory insufficiency. Thus, it is of great importance to assess the safety and therapeutic value of *C. novyi*-NT. Future studies with larger cohorts will be required to verify the protective effect of these antitumor microbiota therapies. An in-depth investigation of the immune alterations in the TME will contribute to uncovering the mechanisms of action of microbiota-centered therapies.

## 7 Conclusions and future perspectives

Intratumoral microbiota is an emerging topic that has been suggested as an important component of the tumor ecosystem. Unlike its intestinal counterpart, the field of intratumoral microbiota is still in its nascency. Compelling evidence has verified the universal presence of intratumoral microbiota in distinct types of cancers. The origin and colonization mechanisms of intratumoral microbiota are poorly understood. Intratumoral microbes may originate from oral and intestinal microbiota. Adjacent normal tissues, mucosal organs and the circulation system are also deemed as potential sources of intratumoral microbiota. Nevertheless, how intratumoral microbes enter tumor cells and the TME is an important question that emerges. The Gal-GalNAc-binding lectin Fap2 of *F. nucleatum* may mediate its entry into Gal-GalNAc-overexpressing cancer cells (39). Whether the interaction between microbes and cancer cells forms the basis for their invasion needs further validation. The comparison of microbial compositions in potential sources (e.g., oral cavity, intestine and mucosal organs) and tumors will be conducive to ascertaining the genuine origin of tumor microbiota in different tumors. *In vivo* tracking of live microbes may assist researches in uncovering the dynamics of their migration process. The integrated 3D quantitative *in situ* intratumoral microbiota imaging method provided the direct evidence of the presence of bacteria within human glioma samples (112). This approach helped to visualize bacteria in their native context in host with single-cell resolution. The 3D *in situ* quantitative imaging technology may be a practical approach to examine the complex interplay among intratumoral microbiota, cancer cells, and the TME. Due to the relatively low microbial biomass within tumors, characterizing tumor microbiota remains a challenge. Undoubtedly, the advances in detection and functional analysis methods (e.g., targeted microbial imaging and multi-omics techniques) will accelerate the discovery of the compositional and functional profiles of intratumor microbial communities. Environmental contamination critically hinders the study of intratumor microbiota. Both experimental and bioinformatic approaches must be developed to exclude inaccurate data. It is intriguing whether the existence of multitudinous microbes within the tumor is a mechanism facilitating their own survival. In addition, the potential impacts of factors such as dietary patterns, drug use, sleep and stress on the composition and local diversity of tumor microbiota are worthy of further study.

The effect of intratumoral microbiota on cancer pathogenesis is an appealing aspect of the host-microbiota interaction. Several significant mechanisms underpinning the involvement of intratumoral microbiota in cancer development have been revealed. Intratumor microbiota can coordinate host genetic mutation and facilitate cancer cell dissemination and metastasis. Specific commensal microbes enhance antitumor immunity through modulation of tumor-associated neutrophils, antitumor M1 macrophages, NK cells and effector T cells, while certain microbes are capable of impelling immunosuppression through diverse mechanisms that affect the function of immunosuppressive cells. Tumor-associated microorganisms dominate cancer cell metabolism and oncogenic signal transduction pathways. Alternative mechanisms mediating the crosstalk between intratumor microbiota and cancer must exist and have yet to be determined. A great deal of work remains to be done to adequately unveil the complex mechanisms through which tumor microbiota affects various aspects of cancer biology. Tumor microbiota-derived metabolites, such as acetate, butyrate and deoxycholic acid, exerted tumor-promoting effects (113–115), which may constitute a crucial mechanism responsible for cancer pathogenesis. Microbe-derived peptides were found to be presented on the surface of melanoma cells, enabling them to be detected by T cells (116). It is inferred that intratumoral microbial peptides widely exist within the TME and thus actuate robust local and systemic immune responses. The role of microbial products in cancer development is therefore a research priority. The interplay between tumor microbiota and the TME has been an important research topic in oncoimmunology. As distinct intratumoral microbes polarize conflictingly different types of the immune responses that either drive or impede cancer progression, tumor microbiota may act in a tumor type- and context-dependent manner as a tumor promoter or suppressor. It is critical to define in which direction the subtle balance between intratumoral microbiota and host immune system is genuinely tipped for specific cancer types. Collectively, more *in vivo* studies will be mandatory to comprehensively characterize the reciprocal interaction between tumor microbiota and the host immune system, which will provide a basis and guidance for further research on this field.

Therapeutic resistance has been a principal obstacle to the success of cancer treatment (117). For instance, a substantial proportion of patients with melanoma and NSCLC demonstrated primary resistance to ICI (118–121). Hence, it is necessary to unearth novel strategies to improve treatment response in cancer patients. Tumor microbiota has the ability to alter therapeutic responsiveness in cancer. On the one hand, intratumoral microorganisms may synergize with anticancer therapeutic, especially immunotherapy, as intratumoral microbiota contributes to increased foreignness of the tumor, strengthening antitumor immune responses (122). On the other hand, intratumoral microorganisms may attenuate the response to anticancer therapies by directly acting on therapeutic agents or lessening antitumor immunity. Harnessing intratumoral microbiota may be helpful in reinforcing the therapeutic effect of existing anticancer regimen. Currently, the mechanisms through which tumor commensal microbes affect the antitumor efficacy of cancer treatment are yet to be fully elucidated. It is of great importance to find out how tumor microbiota protects cancer cells against tumor-killing effects by conventional therapies including chemotherapy, radiotherapy and immunotherapy.

Manipulation of tumor microbiota via targeted reconstitution or improvement of current therapies with supplementation of microbe-derived products may present key elements of future intervention. Several interventional approaches, such as FMT, defined microbial consortium, dietary intervention, antibiotic, prebiotic and probiotic-based approaches, may have the potential to change the composition of gut and tumor microbiota. Although previous clinical trials have positive results, the clinical translation of microbial treatment approaches still faces a myriad of challenges due to the scarcity of ample evidence. It must be noted that the terminal therapeutic effect of microbiota-centered treatments mainly lies on the functionality of tumor microbiota and its impact on the local/systemic immunity. Accordingly, exploring tumor microbiota and its multifaceted modes of action in cancer will offer opportunities for the discovery of new prevention and treatment modalities. Further investigation of microbiota-based treatment strategies aimed at regulating patient tumor microbes in larger patient cohorts represents the ideal next step. The identification and verification of common treatment-associated intratumor microbial signatures in different cancers will greatly advance development of off-the-shelf therapeutic modalities that enable broad applicability across diverse cancer types. Given the heterogeneity of intratumoral microbiota, personalized cancer treatments will be beneficial owing to their high therapeutic efficacy and precise targeting potential. Microbiota modulation has exhibited potential of reinforcing the efficacy of existing cancer treatments and reducing side effects. It is proposed that normalizing the microbiota within the tumor and introducing specific microbes into the TME represent feasible and attractive approaches to optimize the outcome of anticancer therapies (37). Notably, the effects of microbiota-based therapies may vary depending on tumor type, availability of other treatments and the immune status of cancer patients. More clinical studies are needed to determine their real value in assisting cancer intervention. Due to the crosstalk between tumor and gut microbiota, manipulating gut microbiota to treat cancer has been attempted. Nevertheless, it is arduous to disclose the causal mechanisms of microbiota-modulating treatments in complex *in vivo* environments. Further research efforts should be devoted to exploiting microbiota-related knowledge toward the development of improved cancer treatments.

In summary, intratumor microbiota, an integral component of the TME, has emerged as key players in carcinogenesis and cancer development. The potential clinical utility of intratumor microbiota in cancer management has drawn extensive attention in recent years. Thus, intratumor microbiota has become a new frontier in cancer research and a valuable resource of promising targets for personalized cancer care. However, there are still many challenges to be addressed in this field. Progress on all fronts is essential to move intratumor microbiota-related discoveries to the forefront of medical practice.

## Author contributions

MW: Conceptualization, Supervision, Visualization, Writing – original draft. FY: Investigation, Resources, Writing – review & editing. PL: Conceptualization, Supervision, Writing – review & editing.
